# Salicylic acid biosynthesis is enhanced and contributes to increased biotrophic pathogen resistance in *Arabidopsis* hybrids

**DOI:** 10.1038/ncomms8309

**Published:** 2015-06-12

**Authors:** Li Yang, Bosheng Li, Xiao-yu Zheng, Jigang Li, Mei Yang, Xinnian Dong, Guangming He, Chengcai An, Xing Wang Deng

**Affiliations:** 1 Peking-Yale Joint Center for Plant Molecular Genetics and Agro-Biotechnology, State Key Laboratory of Protein and Plant Gene Research, The Peking-Tsinghua Center for Life Sciences, School of Advanced Agricultural Sciences and School of Life Sciences, Peking University, Beijing 100871, China; 2 Department of Molecular, Cellular and Developmental Biology, Yale University, New Haven, Connecticut 06520, USA; 3 Howard Hughes Medical Institute-Gordon and Betty Moore Foundation, Department of Biology, Duke University, Durham, North Carolina 27708, USA; 4 State Key Laboratory of Plant Physiology and Biochemistry, College of Biological Sciences, China Agricultural University, Beijing 100193, China

## Abstract

Heterosis, the phenotypic superiority of a hybrid over its parents, has been demonstrated for many traits in *Arabidopsis thaliana*, but its effect on defence remains largely unexplored. Here, we show that hybrids between some *A. thaliana* accessions show increased resistance to the biotrophic bacterial pathogen *Pseudomonas syringae* pv. *tomato* (*Pst*) DC3000. Comparisons of transcriptomes between these hybrids and their parents after inoculation reveal that several key salicylic acid (SA) biosynthesis genes are significantly upregulated in hybrids. Moreover, SA levels are higher in hybrids than in either parent. Increased resistance to *Pst* DC3000 is significantly compromised in hybrids of *pad4* mutants in which the SA biosynthesis pathway is blocked. Finally, increased histone H3 acetylation of key SA biosynthesis genes correlates with their upregulation in infected hybrids. Our data demonstrate that enhanced activation of SA biosynthesis in *A. thaliana* hybrids may contribute to their increased resistance to a biotrophic bacterial pathogen.

## Abstract (web summary)

The molecular basis for heterosis, the phenomenon whereby hybrid plants show phenotypic superiority to their parents, remains poorly understood. Here, Yang *et al*. show that salicylic acid biosynthesis is enhanced in hybrids of *Arabidopsis thaliana* and correlates with heterosis for pathogen defence.

## Graphical abstract

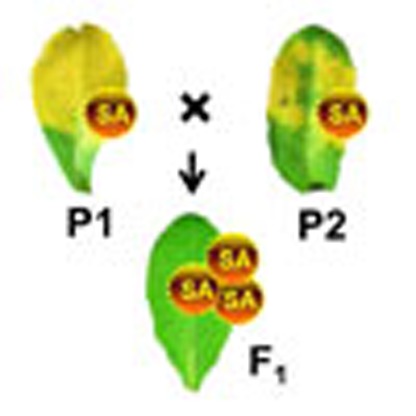

Hybrid vigour, or heterosis, refers to the phenotypic superiority of a hybrid over its parents and has been demonstrated for many traits, such as growth rate, biomass and stress tolerance. Although heterosis has been exploited extensively in crop production for many years, our understanding of its molecular basis is still rudimentary^1^,^2^,^3^,^4^,^5^. Classical theories, including dominance, overdominance and epistasis, have been proposed to explain heterosis from a quantitative genetics perspective^5^,^6^,^7^. These theories have not elucidated the molecular basis of heterosis^6^. Recently, several studies have shed light on potential molecular mechanisms of heterosis in plants. For example, changes in the expression of circadian regulatory genes contribute to biomass heterosis in *Arabidopsis thaliana* by altering circadian rhythms in hybrids^8^. Another study reported the first single overdominant gene, *SINGLE FLOWER TRUSS*, responsible for fruit yield heterosis in tomato^9^. In addition, several studies that surveyed the transcriptomes and epigenomes (including DNA methylomes, small RNAomes and genome-wide distribution of histone modifications) of hybrids and their parental lines in various plant species^1^,^2^,^5^,^10^,^11^ suggesting that both transcriptional and epigenetic variations may play a role in the molecular mechanisms of heterosis.

Natural accessions of *A. thaliana* are largely homozygous due to their selfing property^12^. Hybrids resulting from crosses of two distinct accessions may exhibit heterosis, as demonstrated in *A. thaliana* for biomass^13^,^14^, photosynthetic efficiency^15^, seedling viability^16^, seed number^17^, phosphate uptake^18^ and freezing tolerance^19^,^20^. Two previous studies^21^,^22^ suggested that the inappropriate activation of immune responses in F_1_ hybrids, caused by allelic interactions of NB-LRR immune receptor genes or accelerated cell death genes, was the molecular basis of hybrid necrosis. These studies provided a molecular explanation for hybrid necrosis in which the hybrids exhibited growth defects, but not for the heterotic defence responses in which the hybrids showed normal growth.

Natural variations in the ability of plants to defend against infection are expected because of the significant selective pressure imposed by the pathogens. Plants can be attacked by diverse groups of pathogens, including bacteria, fungi, oomycetes, viruses and nematodes^23^. On recognition of the attacking pathogens, plants can produce immune signals and activate batteries of defence responses^24^,^25^. Salicylic acid (SA) is an immune signal that increases in response to pathogen infection^26^, and this increase often coincides with elevated expression of antimicrobial pathogenesis-related (PR) genes and enhanced disease resistance^27^,^28^. However, mutants or transgenic plants impaired in SA accumulation are unable to trigger efficient defence responses and are hypersusceptible to infection^29^. Moreover, exogenous applications of SA can induce plant resistance to various pathogens^30^.

Two SA biosynthetic pathways exist in plants: one from cinnamate that is catalysed by phenylalanine ammonia lyase, and the other from chorismate that is catalysed by isochorismate synthase (ICS)^28^,^31^. The *A. thaliana* genome contains two *ICS* genes, *ICS1* (also known as *SID2*) and *ICS2*^31^. The function of *ICS1* is predominant, because the total SA level in the *ics1* single mutant after infection is only 5–10% of that in the infected wild type^32^. Other *A. thaliana* mutants that are deficient in SA biosynthesis include *eds5*, *eds1*, *pad4* and the double mutant *cbp60g sard1* (refs 31, 33, 34, 35, 36, 37, 38, 39). *EDS5* (also known as *SID1*) encodes a predicted protein homologous to the multidrug and toxin extrusion family of transporter proteins and is required for exporting SA out of the chloroplasts where it is synthesized^35^,^36^,^37^. In *eds1* and *pad4* mutants, the pathogen-activated expression of *ICS1* and *EDS5* is blocked, suggesting that EDS1 and PAD4 function upstream of ICS1 and EDS5^33^,^38^,^39^. CBP60g and SARD1 are transcription factors that bind to the promoter of *ICS1* and activate its expression. Accordingly, the *cbp60g sard1* double mutant is partially deficient in pathogen-induced SA biosynthesis^40^.

In this study, we show that certain *A. thaliana* crosses have an increased resistance to the biotrophic bacterial pathogen *Pseudomonas syringae* pv. *tomato* (*Pst*) DC3000. We found that the expression levels of several key SA biosynthesis genes were significantly upregulated in hybrids, which ultimately led to higher levels of SA, in both its active free form (SA) and its storage form (SA glucoside, or SAG). Moreover, we show that the increased pathogen resistance in the F_1_ hybrids was compromised when the production of SA was blocked by mutations in *PAD4*. Finally, we discovered that epigenetic modifications were associated with the increased expression of SA biosynthesis genes in hybrids, further supporting our hypothesis that enhanced activation of the SA biosynthesis pathway in hybrids contributes to their increased resistance to this biotrophic bacterial pathogen.

## Results

### Heterosis for biotrophic bacterial defence in *Arabidopsis*

**Figure 1:**
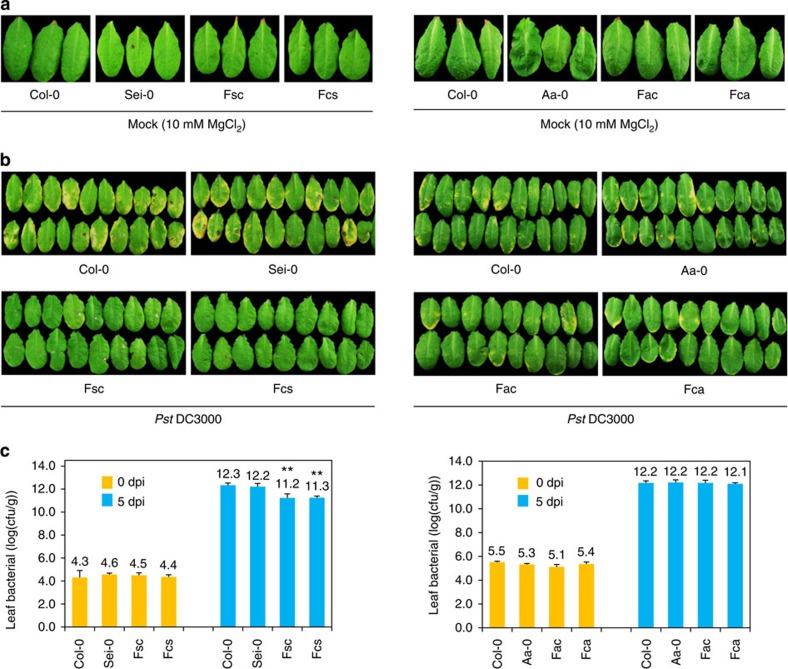
Bacterial defence phenotypes of *Arabidopsis thaliana* F_1_ hybrids and their parents. (**a**) Phenotypes of F_1_ hybrids and their parents 5 dpi with MgCl_2_ (10 mM). Fsc and Fcs, reciprocal F_1_ hybrids, where maternal line is Sei-0 and Col-0, respectively; Fac and Fca, reciprocal F_1_ hybrids, where maternal line is Aa-0 and Col-0, respectively. (**b**) Phenotypes of F_1_ hybrids and their parents 5 dpi with *Pseudomonas syringae* pv. *tomato* (*Pst*) DC3000 (1 × 10^5^ c.f.u. ml^−1^). (**c**) Bacterial titer (log10) of F_1_ hybrids and their parents 0 dpi and 5 dpi with *Pst* DC3000 (1 × 10^5^ c.f.u. ml^−1^). **P<0.01 between hybrids and parents (Student's *t*-test). Bacterial growth is expressed as mean values of viable bacteria per gram of leaf tissue±s.d.. Error bars indicate s.d.. Data are shown as mean±s.d. (*n*=8, n means biological replication).

To investigate whether increased resistance to biotrophic bacterial pathogens exists in certain *A. thaliana* hybrids, we crossed 20 accessions (Supplementary Table 1) reciprocally with *Arabidopsis* ecotype Columbia-0 (Col-0) and evaluated the resulting hybrids and their parents for resistance to the pathogenic bacterium *Pst* DC3000. We investigated leaf phenotypes 1–5 days after pathogen infiltration, and found that the differences between parents and hybrids were most significant at 5 days post infiltration (dpi). Figure 1 shows the leaves of hybrids and their parents from two representative crosses at 5 days after infiltration with *Pst* DC3000 at 1 × 10^5^ colony-forming units (c.f.u.) per ml. We found obvious chlorotic symptoms on the leaves of the three parental accessions (Col-0, Sei-0 and Aa-0) and on the two F_1_ hybrids Aa-0 × Col-0 (represented by Fac) and Col-0 × Aa-0 (represented by Fca) in which Aa-0 or Col-0 was the maternal line, respectively (Fig. 1b). In contrast, chlorotic symptoms were rarely observed on the leaves of the two F_1_ hybrids from the cross between Sei-0 and Col-0 (Sei-0 × Col-0 and Col-0 × Sei-0, represented by Fsc and Fcs, respectively; Fig. 1b).

To further confirm the increased resistance in Fsc and Fcs hybrids, we measured the bacterial titer in leaves at 0 and 5 dpi. As shown in Fig. 1c, while there were no significant differences between parents and hybrids in the initial inoculation amount (0 dpi), the bacterial titer was ∼10-fold lower in hybrids than in their parents at 5 dpi (*P*<0.01, Student's *t*-test), indicating that pathogenic bacterial growth was greatly inhibited in the Fsc and Fcs F_1_ populations. No significant differences in the growth of the pathogenic bacteria were observed between the Fac and Fca hybrids and their parents (Fig. 1c).

Among 20 accessions tested, we found significant heterosis for defence against *Pst* DC3000 (that is, The hybrids were superior to both parents, meaning that pathogenic bacterial growth was significantly inhibited in the F_1_ populations compared with that in either parent (*P*<0.01, Student's *t*-test) in hybrids resulting from crosses between Col-0 and the two accessions Sei-0 and L*er*. No evident heterosis for biotrophic bacterial defence was observed in hybrids resulting from crosses between Col-0 and the other 18 accessions (Supplementary Fig. 1 and Supplementary Table 1). Notably, there were no significant differences in resistance between reciprocal crosses from the same parental pairs, suggesting that there are no obvious maternal effects on heterosis for biotrophic bacterial defence.

A series of studies^21^,^22^,^41^,^42^ reported that some intraspecific hybrids in *A. thaliana* expressed necrosis when grown at 16 °C, but these necrotic phenotypes largely disappeared at 23 °C. This phenomenon was considered to represent negative heterosis for biomass (or hybrid necrosis), as the hybrids showed inferior growth at 16 °C compared with their parents. However, it could also be regarded as a case of positive heterosis for defence, because the hybrids showed an increased resistance to the pathogen *Hyaloperonospora parasitica*^22^,^42^, mediated by increased SA levels^42^ and elevated *PR1* expression^21^. Two studies^21^,^22^, which used QTL mapping and whole-genome transcriptome analyses, showed that hybrid necrosis results from inappropriate activation of the immune system, caused by an interallelic interaction of an NB-LRR immune receptor gene or the accelerated cell death gene *ACD6*. Accordingly, we wondered whether the increased resistance observed in F_1_ hybrids of Col-0 × Sei-0 and Col-0 × L*er* represented cases of hybrid necrosis.

We examined the viability and fertility of F_1_ hybrids from four combinations, Col-0 × Sei-0 and Col-0 × L*er*, which showed heterosis for biotrophic bacterial defence, and Col-0 × Ws and Col-0 × Aa-0, which did not. All F_1_ hybrids were morphologically normal and vigourous at 16 °C (Supplementary Fig. 2a,b), and showed no more cell necrosis than their parents based on trypan blue staining in leaves (Supplementary Fig. 2c). Moreover, compared with their parents, the hybrids exhibiting heterosis for biotrophic bacterial defence (Fsc, Fcs, Flc and Fcl) did not show more highly activated SA pathway genes (*PAD4*, *EDS1*, *SARD1*, *CBP60g*, *ICS1* and *PR1*; Supplementary Fig. 2d). This result was opposite to that found in necrotic hybrids that expressed obvious growth defects and aberrant activation of these genes when grown at 16 °C without pathogen infection. These results suggest that the heterosis we have demonstrated for biotrophic bacterial defence results from a molecular mechanism different from hybrid necrosis.

### SA biosynthesis genes are upregulated in hybrids

To identify genes potentially associated with heterosis for biotrophic bacterial defence, we used mRNA-sequencing (mRNA-seq) to explore the transcriptomes of two *A. thaliana* accessions, Col-0 and Sei-0, and their reciprocal hybrids, Fsc and Fcs, which showed heterosis for *Pst* DC3000 defence. The leaves of 4-week-old hybrid and parental plants grown in short-day conditions were infiltrated with *Pst* DC3000, and 30 leaves were collected from different plants at 1, 2 and 3 dpi. Then, mRNA-seq libraries were constructed and subjected to high-throughput Illumina sequencing. Three independent biological replicates were generated for each sample (Supplementary Table 2). All sequencing reads were aligned to the *A. thaliana* reference genome (Col-0, TAIR10), and a total of ∼900 million genome-matched reads were obtained for 36 libraries (Supplementary Table 3).

**Figure 2:**
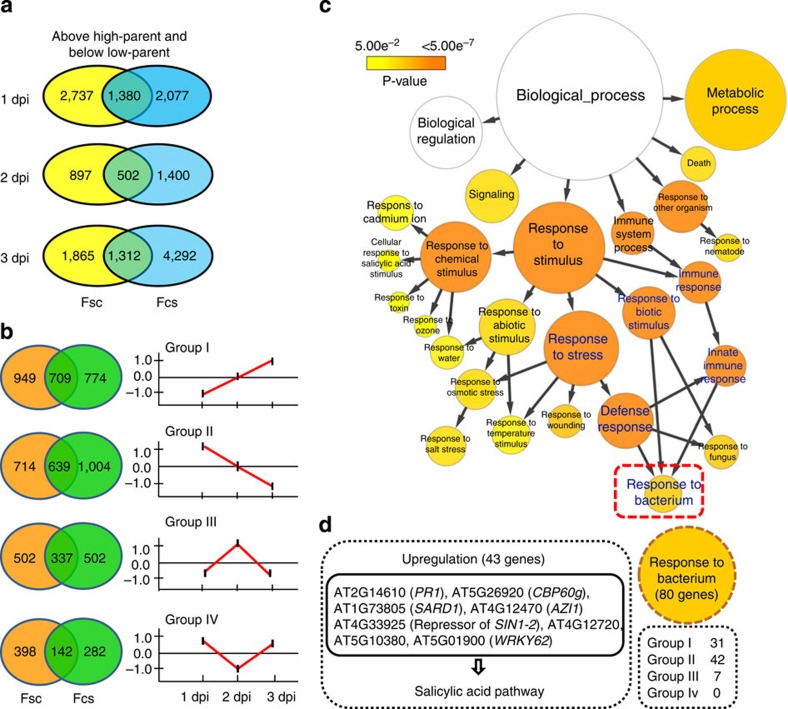
DEGs involved in bacterial defence selected by cluster and functional classification analyses. (**a**) Venn diagram showing the number of ‘above-high parent' and ‘below-low parent' genes in each *Arabidopsis thaliana* hybrid at three time points. (**b**) Venn diagram showing the number of genes (selected from (**a**)) in each Cluster Analysis Group in F_1_ hybrids. Group I: expression continuously increased; group II: expression continuously decreased; group III: expression increased first and then decreased; and group IV: expression decreased first and then increased. (**c**) Enrichment of selected GO categories for genes selected from (**b**). The biological process with false discovery rate (adjusted *P*<0.05, Student's *t*-test) is shown. (**d**) Some genes in the salicylic acid pathway, categorized as ‘response to bacterium' by GO analysis, were upregulated in F_1_ hybrids.

At each time point after inoculation, we identified differentially expressed genes (DEGs) between parents and hybrids (Supplementary Figs 3, 4 and 5). We identified between 2,506 and 5,577 DEGs that showed non-additive actions in reciprocal hybrids. The non-additive group was subdivided into four distinct modes: above-high parent, high parent, low parent and below-low parent (Supplementary Fig. 3c,d). A total of 3,194 DEGs that showed above-high parent or below-low parent expression in both hybrids (Fig. 2a; Supplementary Data 1) were chosen for further analysis. These 3,194 genes were further classified into four groups based on expression changes across the three time points: expression continuously increased; expression continuously decreased; expression increased and then decreased; and expression decreased and then increased. On the basis of these criteria, 1,827 genes showing the same expression patterns between reciprocal hybrids were selected for Gene Ontology (GO) analysis (Fig. 2b; Supplementary Data 1). The most significantly enriched GO term was ‘response to stimulus' (*P*<5.00*e*^−7^, Hypergeometric test; Fig. 2c). A set of 80 genes termed ‘response to bacterium' (Fig. 2d; Supplementary Data 1) seemed the most relevant to the goal of this study and was studied further. We compared the expression levels of these 80 genes between hybrids and parents, and found that the genes with the most significant change in expression between parents and hybrids were enriched in the SA pathway. These included *CBP60g* and *SARD1*, which encode the transcriptional activators of *ICS1*, and *PR1*, the most commonly used marker gene for SA signalling (Fig. 2d).

**Figure 3:**
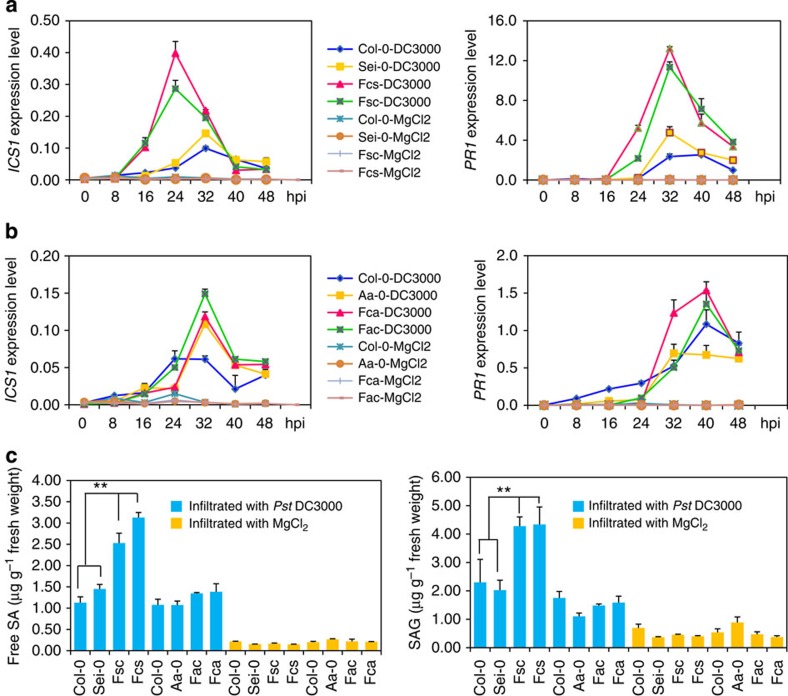
Free SA and SA glycoside levels in *Arabidopsis thaliana* F_1_ hybrids and their parents. (**a**) qPCR analyses of *ICS1* and *PR1* expression in F_1_ hybrids and parents of Col-0 × Sei-0 every 8 h post infiltration (hpi) up to 48 hpi with *Pseudomonas syringae* pv. *tomato* (*Pst*) DC3000 at 1 × 10^5^ c.f.u. ml^−1^ and MgCl_2_. Fsc and Fcs, reciprocal F_1_ hybrids, where maternal line is Sei-0 and Col-0, respectively. (**b**) qPCR analyses of *ICS1* and *PR1* expression in F_1_ hybrids and parents of Col-0 × Aa-0 1 every 8 h post infiltration (hpi) up to 48 hpi with *Pst* DC3000 at 1 × 10^5^ c.f.u. ml^−1^. Fac and Fca, reciprocal F_1_ hybrids, where maternal line is Aa-0 and Col-0, respectively. Data are standardised for abundance of *Actin* transcript. (**c**) Free SA and SAG levels in F_1_ hybrids and parents of two crosses 1 dpi with *Pst* DC3000 at 1 × 10^5^ c.f.u. ml^−1^. The results are a representative of three biological repetitions. Error bars indicate s.d.. Data are shown as mean±s.d. (*n*=3, n means technical replication). **P<0.01 between hybrids and parents (Student's *t*-test).

SA is a well-known hormone important for plant defence^28^,^29^. That genes involved in the SA pathway changed significantly in hybrids relative to their parents suggests that enhanced activation of the SA pathway may be related to the increased resistance to *Pst* DC3000 in *A. thaliana* hybrids. To test this hypothesis, we extended our expression analysis by performing detailed time–course experiments. Leaf samples of hybrids and parents were collected every 8 h after infiltration up to 48 h and subjected to real-time quantitative reverse transcription PCR (qRT–PCR). As shown in Figs 3 and 5 and Supplementary Fig. 6, expression of *PR1*, *PAD4*, *EDS1*, *SARD1* and *CBP60g* was significantly upregulated in the F_1_ hybrids of Col-0 × Sei-0 compared with their parents, and expression of these genes peaked in the hybrids 8 h earlier than in their parents. No expression changes were observed for these genes in the F_1_ hybrids of Col-0 × Aa-0 (Fig. 3b; Supplementary Fig. 7), which is consistent with the lack of evident heterosis for *Pst* DC3000 defence in these hybrids (Fig. 1). These results imply that the upregulation of key genes in the SA biosynthesis pathway in F_1_ hybrids may play a role in the increased resistance of hybrids to biotrophic bacterial pathogen.

### More SA accumulates in hybrids after *Pst* DC3000 inoculation

In *A. thaliana*, pathogen-induced biosynthesis of SA starts from chorismate is catalysed by ICS1^31^,^32^. On the basis of our mRNA-seq data, the expression of *ICS1* at 1 dpi was 10-fold higher in F_1_ hybrids than in parents, suggesting that SA biosynthesis is more active in F_1_ hybrids than in their parents after pathogen infection. To further test this idea, we analysed expression of *ICS1* in F_1_ hybrids of Col-0 × Sei-0 and Col-0 × Aa-0 relative to their respective parents after infiltration with *Pst* DC3000 or 10 mM MgCl_2_ (control). As shown in Fig. 3a,b, the expression level of *ICS1* showed a greater and more rapid increase in the reciprocal F_1_ hybrids of Col-0 × Sei-0, but not in the F_1_ hybrids of Col-0 × Aa-0, compared with their respective parents.

Next, we determined the SA levels in F_1_ hybrids and their parents after infiltration with *Pst* DC3000. As shown in Fig. 3c, at 1 dpi, more SA (including free SA and SAG) had accumulated in the F_1_ hybrids Fsc and Fcs than in their parents (*P*<0.01, Student's *t*-test). No significant changes in SA accumulation were observed in the F_1_ hybrids Fac and Fca relative to their parents. Altogether, our data indicate that increased SA accumulation may be responsible for increased resistance to biotrophic bacterial pathogens in the F_1_ hybrids Fcs and Fsc.

An important defence response induced by SA is the triggering of PR gene expression, especially *PR1*^43^. An examination of *PR1* expression showed that *PR1* was significantly upregulated in the reciprocal F_1_ hybrids Fsc and Fcs relative to their parents, whereas no obvious changes in *PR1* expression were found in the reciprocal F_1_ hybrids Fac and Fca relative to their parents (Fig. 3a,b). These results indicate that increased SA accumulation, possibly due to enhanced activation of the SA biosynthesis pathway, may be involved in increased resistance to biotrophic bacterial pathogens in *A. thaliana* hybrids.

### Blocking SA biosynthesis compromises heterosis in hybrids

**Figure 4:**
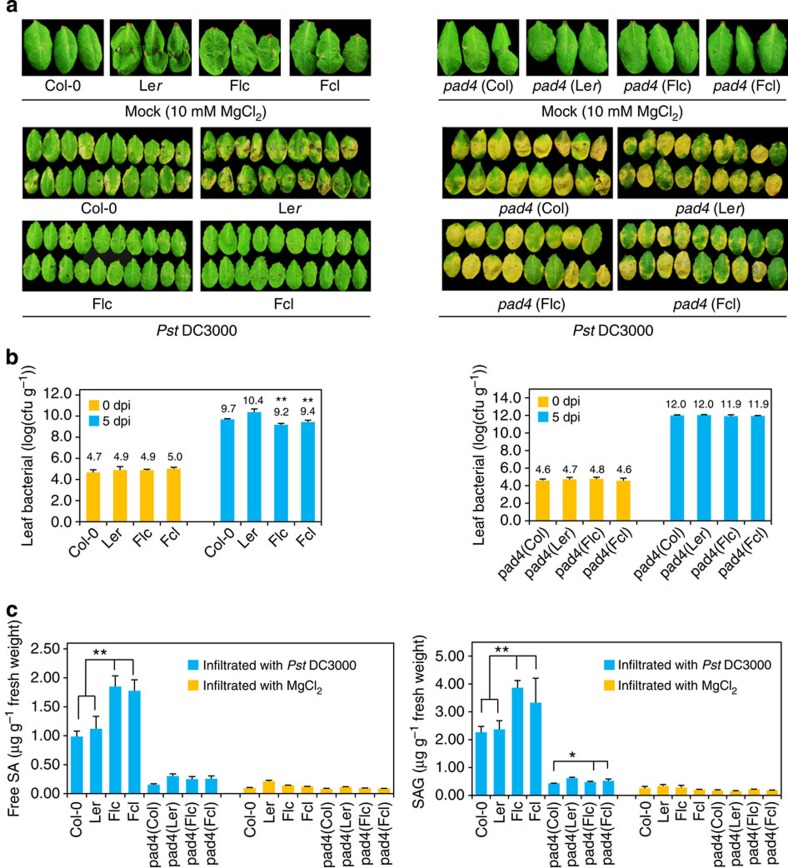
Increased resistance to the biotrophic bacterial pathogen was compromised in F_1_ hybrids of *pad4* mutants. (**a**) Phenotypes of Col-0 × L*er* and *pad4* (Col) × *pad4* (L*er*) parental lines and their respective F_1_ hybrids 5 dpi with MgCl_2_ (10 mM) or *Pseudomonas syringae* pv. *tomato* (*Pst*) DC3000 (1 × 10^5^ c.f.u. ml^−1^). Flc and Fcl, reciprocal F_1_ hybrids, where maternal line is L*er* and Col-0, respectively. (**b**) Bacterial titer (log10) of Col-0 × L*er* and *pad4* (Col) × *pad4* (L*er*) parental lines and their respective F_1_ hybrids 5 dpi with *Pst* DC3000 (1 × 10^5^ c.f.u. ml^−1^). Bacterial growth is expressed as mean values of viable bacteria per gram of leaf tissue±s.d. Error bars indicate s.d.. Data are shown as mean±s.d. (*n*=8, n means biological replication). (**c**) Free SA and SA glycoside (SAG) levels in Col-0 × L*er* and *pad4* (Col) × *pad4* (L*er*) parental lines and their respective F_1_ hybrids 1 dpi with *Pst* DC3000 at 1 × 10^5^ c.f.u. ml^−1^. The results are a representative of three biological repetitions. Error bars indicate s.d.. Data are shown as mean±s.d. (*n*=3, *n* means technical replication). *P<0.05 and **P<0.01 between hybrids and parents (Student's *t*-test).

To substantiate the role of SA in heterosis for biotrophic bacterial defence, we asked whether blocking the SA biosynthesis pathway could compromise the heterotic phenotypes of F_1_ hybrids. For this purpose, we chose *PAD4* to examine as a key gene in the SA biosynthesis pathway^44^ that is mutated in more than one *A. thaliana* ecotype. PAD4 is a lipase-like protein that functions upstream of the SA biosynthesis pathway to control defence responses in *A. thaliana*^33^. Compared with wild type, *pad4* mutants display enhanced susceptibility to two virulent *P. syringae* pathogens (*Pst* DC3000^43^ and *P. syringae* pv. *maculicola* ES4326^45^,^46^), resulting from the reduced synthesis and accumulation of SA and SAG^33^,^38^. A *pad4* mutant line in the L*er* background (*pad4-2*)^44^ and a T-DNA insertion line in the Col-0 background (SALK_089936) were used to generate reciprocal F_1_ hybrid lines. We first confirmed that expression of *PAD4* was downregulated in the two *pad4* parental lines (Supplementary Fig. 8). In the absence of a *pad4* mutation, the F_1_ hybrids of Col-0 and L*er* (Flc and Fcl) had significantly increased resistance to biotrophic bacterial invasion (*P*<0.01, Student's *t*-test; Fig. 4a,b), and significant greater accumulation of both free SA and SAG (*P*<0.01, Student's *t*-test; Fig. 4c) relative to their parents. For *pad4* mutants, both the parental lines (*pad4* (Col) and *pad4* (L*er*)) and their reciprocal F_1_ hybrids (*pad4* (Flc) and *pad4* (Fcl)) showed greater susceptibility to *Pst* DC3000 than their wild-type counterparts (Fig. 4a,b). Moreover, the heterosis for biotrophic bacterial defence in both reciprocal F_1_ hybrids was greatly compromised, and no significant differences in symptoms or bacterial titers were observed among the hybrids and parents (Fig. 4a,b). In addition, there were no significant differences in SA accumulation in the F_1_ hybrids compared with their parents (Fig. 4c). Notably, SA levels were reduced to control levels (treated with 10 mM MgCl_2_) in both parents and hybrids (Fig. 4c). We then tested the expression profiles of *ICS1* and *PR1*, which act downstream of *PAD4* in the SA biosynthesis pathway. At 1 dpi, both genes were upregulated in F_1_ hybrids Flc and Fcl, but not in F_1_ hybrids *pad4* (Flc) and *pad4* (Fcl) (Supplementary Fig. 9). These data further indicate that the increased level of SA resulting from enhanced activation of the SA biosynthesis pathway in hybrids plays an essential role in heterosis for biotrophic bacterial defence.

### H3Ac of SA biosynthesis genes increased in hybrids

**Figure 5:**
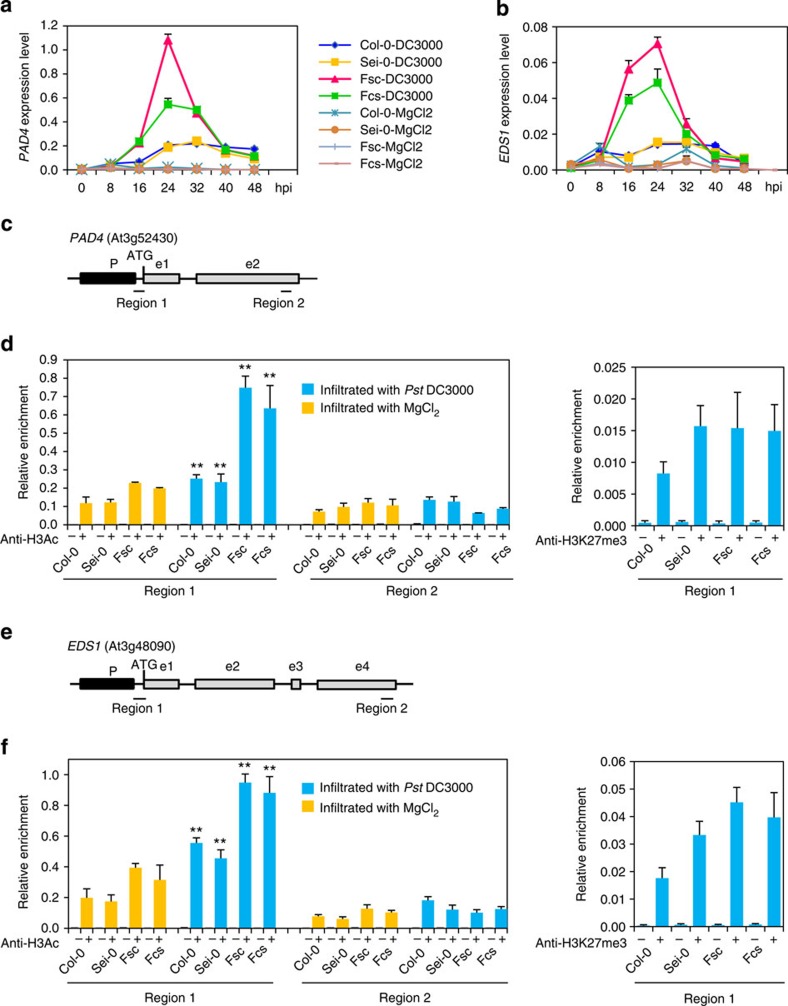
Increased H3 acetylation correlates with altered expression of *PAD4* and *EDS1* in F_1_ hybrids. (**a**,**b**) qPCR analyses of *PAD4* and *EDS1* expression in F_1_ hybrids and parents of Col-0 × Sei-0 every 8 h post infiltration (hpi) up to 48 hpi with *Pseudomonas syringae* pv. *tomato* (*Pst*) DC3000 at 1 × 10^5^ c.f.u. ml^−1^ or MgCl_2_. Fsc and Fcs, reciprocal F_1_ hybrids, where maternal line is Sei-0 and Col-0, respectively. Data are standardized for abundance of *Actin* transcript. (**c**,**e**) Regions of *PAD4* and *EDS1* used for ChIP–qPCR assays. (**d**,**f**) ChIP–qPCR analyses of promoter fragments (region 1) and exon fragments (region 2) of *PAD4* and *EDS1* in F_1_ hybrids and their parents using anti- H3Ac antibody at 1 dpi with *Pst* DC3000 or MgCl_2_, and ChIP–qPCR analyses of region 1 of *PAD4* and *EDS1* in F_1_ hybrids and their parents using anti-H3K27me3 antibody at 1 dpi with *Pst* DC3000. Error bars indicate s.d.. **P<0.01 between infiltrated with *Pst* DC3000 and MgCl_2_ of respective samples (Student's *t*-test). ChIP values were normalized to their respective DNA inputs. Data are shown as mean±s.d. The results are a representative of three biological repetitions. Error bars indicate s.d.. Data are shown as mean±s.d. (*n*=3, n means biological replication).

Several reports recently showed that chromatin modifications may play important roles in hybrid vigour^10^,^47^. Chromatin modifications have also been suggested to play a role in plant defence responses by regulating gene expression^48^,^49^. We thus asked whether chromatin modifications might be involved in heterosis for biotrophic bacterial defence in *Arabidopsis*. We examined the histone modifications of *PAD4*, *EDS1*, *CBP60g* and *SARD1* in both hybrids and their parents using chromatin immunoprecipitation (ChIP) followed by quantitative real-time PCR (qRT-PCR). Among the standard histone modifications, acetylation of histone H3 (H3Ac) is associated with active chromatin and transcription^50^,^51^, and the trimethylation of H3K27 (H3K27me3) is associated with repressed transcription^52^,^53^. Therefore, we performed ChIP assays using anti-H3Ac and anti-H3K27me3 antibodies against chromatin extracted from leaves infiltrated with *Pst* DC3000 or MgCl_2_ (as a control). The precipitated DNA was purified and analysed by quantitative real-time PCR (qRT-PCR) to assess the chromatin states of the four gene promoters. For *PAD4* and *EDS1*, a region close to the translation initiation codon (ATG) was examined using anti-H3Ac and anti-H3K27me3, and an exon region downstream of the ATG was examined using anti-H3Ac as a control (Fig. 5c,e). As expected, there was a significant increase in H3Ac at the promoter regions of *PAD4* and *EDS1* in the reciprocal F_1_ hybrids relative to the parents after infiltration with *Pst* DC3000 or MgCl_2_, and the increase was higher after infiltration with *Pst* DC3000 than with MgCl_2_ (Fig. 5d,f). There was no significant difference in the level of H3K27me3 in these regions between reciprocal F_1_ hybrids and parents after infiltration with *Pst* DC3000 (Fig. 5d,f). These results are consistent with the upregulation of these two genes in the F_1_ hybrids (Fig. 5a,b). Similar patterns of H3Ac and H3k27me3 were also observed for *CBP60g* and *SARD1* (Supplementary Fig. 6). We performed the same experiment using the Col-0 × Aa-0 cross, which had no heterosis for biotrophic bacterial defence, and found that there was no significant increase in H3Ac at the promoter regions of *PAD4*, *EDS1*, *SARD1* and *CBP60g* in the reciprocal F_1_ hybrids relative to the parents after infiltration with MgCl_2_ or *Pst* DC3000, which is consistent with the no significant difference in gene expression between these hybrids and their parents (Supplementary Fig. 7).

Recently, changes in DNA methylation have been correlated with transcription level changes in hybrids, suggesting that DNA methylation plays a role in heterosis for biomass^10^,^11^. To further investigate whether DNA methylation contributes to the increased pathogen resistance in hybrids, we used bisulphite sequencing to examine the methylation levels of CG, CHG and CHH (where H=A, T or C) in the promoters or gene body regions of *PAD4*, *EDS1*, *ICS1*, *SARD1* and *CBP60g* in hybrids and their parental lines after infiltration with *Pst* DC3000^54^. The selected regions were chosen based on a previous whole-genome DNA methylation profiling study in *A. thaliana*^55^. Degenerate primers flanking these regions were used to amplify bisulphite-treated DNA, and the PCR products were subsequently cloned and sequenced. When methylation levels of CG, CHG and CHH sites were calculated and compared between F_1_ hybrids and parents, we found that the total methylation levels in the F_1_ hybrids for all tested regions were at mid-parent levels (Supplementary Fig. 10 and Supplementary Data 2), suggesting that DNA methylation may not play a role in heterosis for biotrophic bacterial defence in our tested accessions.

**Figure 6:**
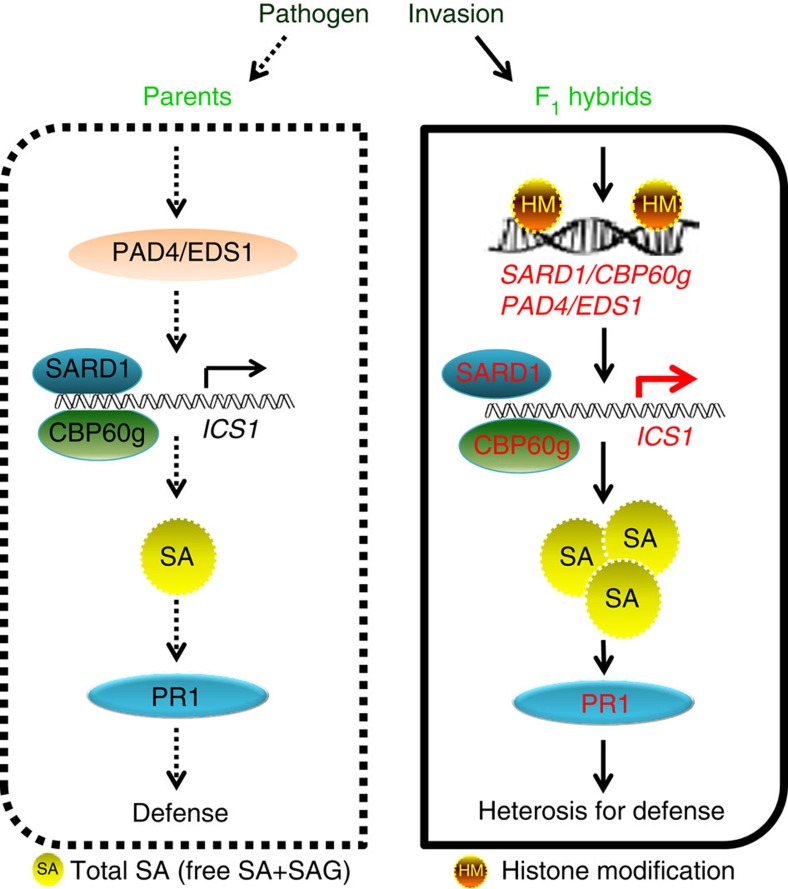
Working model of enhanced SA biosynthesis in hybrids. SA biosynthesis is activated in *Arabidopsis thaliana* F_1_ hybrids and their parents in response to pathogen invasion as a defence strategy, but is not observed in the absence of pathogens. Genes involved in SA biosynthesis were expressed at higher levels in F_1_ hybrids, possibly due to altered histone modifications (such as increased H3Ac). As a consequence, higher levels of SA accumulated in the F_1_ hybrids, which we propose plays an important role in the increased resistance of hybrids to biotrophic bacterial pathogens.

Our data demonstrate that, in response to pathogen invasion, SA biosynthesis was activated, which has not been observed in the absence of pathogens, in both F_1_ hybrids and their parents as a defence strategy. However, the key genes involved in SA biosynthesis were expressed at higher levels in the F_1_ hybrids than in their parents, possibly through regulation of histone modifications, such as H3 acetylation. Consequently, higher levels of SA, which plays an important role in resistance to biotrophic bacterial pathogens in *A. thaliana*, are produced in F_1_ hybrids (Fig. 6).

## Discussion

In this study, we identified certain F_1_ hybrids manifesting significant heterosis (superiority to both parents) for biotrophic bacterial resistance in *A. thaliana*. We compared genome-wide gene expression profiles of hybrids showing heterosis with those of their parents and found that the expression of key SA biosynthesis genes was significantly upregulated in both reciprocal F_1_ hybrids compared with either parent (Figs 3 and 5; Supplementary Fig. 6). This indicated that SA accumulation might be increased in hybrids, which was confirmed by direct determination of SA levels in hybrids and parents after bacterial inoculation (Fig. 3). The increased pathogen resistance in hybrids was compromised by *pad4*, as shown in the F_1_ hybrids of *pad4* (L*er*) and *pad4* (Col) (Fig. 4), indicating that SA is required for heterosis for biotrophic bacterial defence. Moreover, the upregulated expression of key SA biosynthesis genes was associated with higher levels of H3Ac in the promoters of these SA-related genes in the F_1_ hybrids than in the parents (Fig. 5 and Supplementary Fig. 6). As H3 acetylation is associated with active gene expression, this increase suggests that epigenetic regulation of the SA biosynthesis pathway may play an important role in the increased resistance to biotrophic bacterial pathogens in hybrids.

A previous study^56^ showed that Ws-0 is insensitive to the bacterial flagellin flg22, whereas Ws-0 × Col-0 (flg22 sensitive) exhibited sensitive phenotypes in F_1_ seedlings. The corresponding dominant locus was further segregated and later found to encode the flagellin receptor FLS2 (refs 57, 58). These studies explain why hybrid crosses between Col-0 and Ws in our study did not show heterosis for biotrophic bacterial defence (Supplementary Fig. 1).

On treatment with SA^59^,^60^, β-aminobutyric acid^61^ or various other natural or synthetic compounds^62^, plants acquire enhanced resistance to a broad spectrum of pathogens, a phenomenon called ‘inducible defence'. The various induced resistance phenotypes are associated with an enhanced capacity for more rapid and effective activation of cellular defence responses and the expression of various defence-related genes, which were induced only after pathogen attack^59^,^60^,^63^. This physiological condition, in which plants are able to better or more rapidly mount defence responses to biotic or abiotic stress, is called the ‘primed state'^64^. As revealed in our study, the key genes involved in the SA biosynthesis pathway (*PAD4*, *EDS1*, *SARD1*, *CBP60g* and *ICS1*) were all significantly upregulated in F_1_ hybrids relative to their parents after inoculation with the pathogen (Figs 3 and 5; Supplementary Fig. 6), eventually leading to increased SA accumulation in hybrids (Fig. 3c). Moreover, the expression peak for all of these genes was 8 h earlier in hybrids than in their parents, and this faster activation was only induced under pathogen invasion (Figs 3 and 5; Supplementary Fig. 6). These observations indicate that F_1_ hybrids responded more efficiently to pathogen attack than did their parents by expressing SA pathway genes more quickly and at higher levels. Therefore, F_1_ hybrids showed a primed state under pathogen attack, which might be an important step towards heterosis for biotrophic bacterial defence in *A. thaliana*.

The mechanistic basis of gene priming is poorly understood until now. Histone modifications such as H3K4 trimethylation and H3 acetylation are systemically set following treatment with the SA analogue benzothiadiazole^65^. However, the primed genes were not activated or were activated to a limited extent, even with altered histone modifications. Instead, these changes in histone modifications may serve as a memory of the primary treatment, which is then associated with an amplified reaction to the following stress (water infiltration)^65^. In our study, H3 acetylation levels in the promoter regions of the key SA biosynthesis genes were higher in F_1_ hybrids than in their parents (Fig. 5; Supplementary Fig. 6) even after mock (10 mM MgCl_2_) treatment, and this difference increased further after inoculation with bacterial pathogen. Moreover, the higher level of histone modifications in the F_1_ hybrids under MgCl_2_ inoculation did not induce expression of these genes (Fig. 5; Supplementary Fig. 6), an observation reminiscent of the findings reported in the abovementioned study^65^. Accordingly, we speculate that hybridization might induce a primed state characterized by enhanced histone acetylation in the promoter regions of these SA biosynthesis genes, which then enables a more rapid and stronger activation of defence-related genes on pathogen invasion.

A previous study^66^ showed that promoter insertion/deletion (INDEL) polymorphisms were highly correlated with differential gene expression between rice hybrids and their parents, which might contribute to the biomass heterosis. Therefore, we also sequenced the 2-kb regions upstream of the ATG codons of the key SA biosynthesis genes in different *A. thaliana* ecotypes. Surprisingly, we found an identical 612-bp INDEL in the promoter of *CBP60g* in nine ecotypes (Aa-0, No-0, Nd-0, Est-0, C24, Co-1, Gr-1, Ha-0, Dr-0), and none of the F_1_ crosses of Col-0 with these nine ecotypes showed increased resistance to the biotrophic bacterial pathogen. The specific relationship between this INDEL and the differential expression level of genes in hybrids and parents remains to be explored. It has been proposed^20^ that heterosis for biomass and for freezing tolerance were not genetically related in *A. thaliana*. In this study, our data suggest that there was no direct relationship between heterosis for biomass and for biotrophic bacterial resistance. The F_1_ hybrids from two combinations (Col-0 & C24 and L*er* & Col-0), which exhibit heterosis for biomass^11^,^14^, show no heterosis for biotrophic bacteria defence in our study (Supplementary Fig. 11). The F_1_ hybrids from two other combinations (Col-0 & Sei-0 and Col-0 & L*er*) exhibit heterosis for biotrophic bacteria defence, but show no (crosses between Col-0 and Sei-0) or only weak (crosses between Col-0 and L*er*) heterosis for biomass (Supplementary Table 4). Taken together, these studies suggest that different molecular mechanisms account for different heterotic traits.

## Methods

### Plant materials

The *A. thaliana* accessions Aa-0 (N934), Est-0 (N1148) and Sei-0 (N1504) were obtained from the Nottingham *A. thaliana* Stock Centre. All other accessions, WS-2 (CS76631), L*er*-1 (CS6928), C24 (CS28127), Co-1 (CS76468), Gr-1 (CS76496), Ha-0 (CS76500), Dr-0 (CS76475), Kil-0 (CS76526), Kro-0 (CS76533), Bch-1 (CS76444), Db-1 (CS76471), Hs-0 (CS76515), An-1 (CS76435), La-0 (CS76538), Lip-0 (CS76542), No-0 (CS24239) and Nd-0 (CS6803), were obtained from the *A. thaliana* Biological Resource Center. Crosses were performed by dissecting immature flowers before anther dehiscence and applying pollen to the exposed pistils. F_1_ hybrid lines were generated by crossing the indicated parental lines. F_1_ hybrids of *pad4* mutants were generated by crossing a T-DNA insertion line in the Col-0 background (SALK_089936) with *pad4-2* in the L*er* background^38^.

### Growth conditions

Plants were grown on Murashige and Skoog plates containing 1% sucrose at 23 °C under white light conditions (100 μmol m^−2^ s^−1^; 16-h light/8-h dark). Plants for pathogen inoculation were grown under short-day conditions (8-h light) and leaves from 4-week-old plants were used. For other assays, 6-week-old plants grown at 16 °C under long-day conditions (16-h light) were used. All plants were grown at the controlled temperature (16° or 23°±0.1°C) with 65% relative humidity. Chambers were illuminated using a 1:1 mix of Cool White and Gro-lux wide-spectrum fluorescent lights with a fluence rate of 125–175 μmol m^−2^ s^−1^.

### RNA extraction and qRT–PCR

Leaves from 4-week-old plants were infiltrated with *Pst* DC3000 (1 × 10^5^ c.f.u. ml^−1^) or with 10 mM MgCl_2_ (mock) and collected at different time points. At least five leaves from different plants were pooled in each sample for qRT–PCR. Leaves were ground to a powder in liquid nitrogen, and total RNA was extracted using the RNeasy Plant Mini kit (Qiagen) with an On-Column DNase I digestion treatment. Spectrophotometric and gel electrophoretic analyses were performed to detect RNA quality. To synthesize cDNA, 1 μg of RNA was used in the SuperScript III First-Strand Synthesis System (Invitrogen). Real-time qPCR analysis was performed using Power SYBR Green PCR Master Mix (Applied Biosystems) on a Bio-Rad CFX96 real-time PCR detection system. Each experiment was repeated with three independent samples, and qRT–PCR reactions were performed in three technical replicates for each sample. All primers used are listed in Supplementary Table 5.

### mRNA-seq and data analysis

At least 30 leaves from different 4-week-old plants infiltrated with *Pst* DC3000 (1 × 10^5^ c.f.u. ml^−1^) were collected at 1, 2 and 3 days after infiltration. Total RNA was extracted as described above and was treated with RNase-free DNase I (New England Biolabs) to remove any contaminating genomic DNA. Total RNA (1–10 μg) was used for the construction of each mRNA-seq library. mRNA was extracted from total RNA using Dynabeads oligo(dT) (Invitrogen Dynal) following the manufacturer's directions. First- and second-strand cDNA were generated using Superscript II reverse transcriptase (Invitrogen) and random hexamer primers. Double-stranded cDNA was fragmented by nebulization and used for mRNA library construction following the standard Illumina protocol. Barcodes were added to each mRNA-seq library, and all libraries were sequenced using the HiSeq 2000 sequencing system according to the manufacture's instruction (Illumina). TopHat and Cufflink software packages were used for the mRNA-seq data analysis to identify DEGs. The BiNGO plugin of Cytoscape software was used for GO analysis. SAS software was used for significant difference analysis.

After sequencing adapters and low-quality reads were removed, sequencing reads were mapped to the TAIR 10 *A. thaliana* reference genome by TopHat (http://tophat.cbcb.umd.edu/) using default parameters. The TopHat results were exported into Cufflink software so that the sequencing reads could be assembled into known transcripts based on the *A. thaliana* gene model annotation of TAIR10. The abundance of assembled transcripts was also calculated in fragments per kilobase of exon model per million mapped fragments (FPKM). Following Cufflink, Cuffmerge software was used to merge the assembled transcripts from each sample into one file using default parameters. Finally, transcript abundance profiling was undertaken by Cuffdiff software using a Poisson fragment distribution and a false discovery rate (FDR) <0.05 as the two default parameters. Cufflink, Cuffmerge and Cuffdiff are from the Cufflink software package (http://cufflinks.cbcb.umd.edu).

To classify genes into expression patterns, non-additive expression levels in hybrids were identified by comparing the expression in hybrids with the mid-parent values; genes with *P*<0.05 (Student's *t*-test) were considered to be non-additively expressed, and genes with *P*>0.05 (Student's *t*-test) were considered to be additively expressed. Similarly, for genes that were differentially expressed in the parents, if expression in the hybrids was significantly different from the high parent but not from the low parent, then they were classified as ‘low parent expression'; if expression in the hybrids was significantly different from the low parent but not from the high parent, then they were classified as ‘high parent expression'.

GO results were extracted from the TAIR10 gene annotation, and a functional enrichment analysis was performed using the BiNGO plugin of Cytoscape (http://www.psb.ugent.be/cbd/papers/BiNGO/Home.html). Pearson product–moment correlation coefficients were calculated using R software.

### Bacterial inoculation and determination of bacterial growth

*Pst* DC3000 was grown at 28 °C in King's B medium (10 mg ml^−1^ protease peptone, 1.5 mg ml^−1^ K_2_HPO_4_, 15 mg ml^−1^ glycerol)^67^ supplemented with 25 μg ml^−1^ rifampicin. Mature, fully expanded leaves of 4-week-old plants were infected with suspensions of bacterial cells in 10 mM MgCl_2_ by pressing a 1-ml syringe (without a needle) against the abaxial side of the leaves and forcing the suspension through the stomata into the intercellular spaces. The bacterial dose was 1 × 10^5^ c.f.u. cm^−2^ leaf area (equivalent to OD_600_=0.0002).

Five days after inoculation, the degree of bacterial growth in plant leaves was determined by harvesting 16–24 infected leaves per sample (approximately eight plants), and a 0.28-cm^2^ leaf disk was cut from each leaf with a no. 2 cork borer. Disks were put into a microcentrifuge tube containing 1 ml of 10 mM MgCl_2_ and ground with a plastic pestle. This material was diluted, and 60-μl samples were spread on King's B plates containing 25 μg ml^−1^ rifampicin. Plates were incubated for 2 days at 28 °C. Eight replicate samples per genotype were assayed to obtain means and s.d., which were determined from the logarithm of the number of c.f.u.  cm^−2^.

### Determination of endogenous levels of SA and SAG

Mature leaves of 4-week-old plants were infected with *Pst* DC3000 at a dose of 1 × 10^5^ c.f.u. cm^−2^ leaf area (equivalent to OD_600_=0.0002) or mock infected with 10 mM MgCl_2_. At 1 dpi, samples were collected (at least 0.2 g tissue per sample, from approximately eight plants) and frozen in liquid nitrogen. SA and SAG were extracted and quantified from ∼200 mg of tissue per sample using high-performance liquid chromatography analysis on an ARH-601 organic acids column (100 mm × 6.5 mm; Transgenomic Inc., Omaha, NE, USA) run at 55 °C in 0.01 N H_2_SO_4_ with a flow rate of 0.6 ml min^−1^. Three replicates were taken for each data point. Statistical analyses were performed using Student's *t*-test of the differences between the two means.

### ChIP

About 2 g materials were cross-linked with 1% formaldehyde in a vacuum for 35 min, and were then ground to powder in liquid nitrogen. The chromatin complexes were isolated and sonicated, and were then incubated with anti-acetyl-Histone H3 antibody and anti-trimethyl-Histone H3 (Lys27) antibody. 10 μg of anti-AcH3 (Upstate; 06-599) and anti-H3K27me3 (Millipore; 07-449) antibodies^49^,^68^ were used in a 10-μl volume for immunoprecipitation. An equal amount of sample without antibody was used as a mock control. The precipitated DNA was recovered and analysed by qPCR with Power SYBR Green PCR Master Mix (Applied Biosystems) using specific primers listed in Supplementary Table 5. Each ChIP value was normalized to its respective input DNA value. All ChIP–qPCR experiments were independently performed in triplicate.

### Genomic DNA extraction and bisulphite sequencing

Four-week-old plants were grown under short-day conditions and infiltrated with *Pst* DC3000. Leaves were collected at 1 dpi and genomic DNA was extracted using the DNeasy Plant Mini kit (Qiagen). Then, ∼500 ng genomic DNA was used for bisulphite conversion with the EZ DNA Methylation-Gold kit (ZYMO Research) according to the manufacturer's instructions. Bisulphite-treated DNA (1 μl) was then amplified by PCR in a 25-μl reaction using ZymoTaq DNA polymerase (ZYMO Research) and degenerate primers (Supplementary Table 5). PCR products were resolved on a 1% agarose gel, and then excised, purified and cloned into a pGEM-T vector (Promega) for sequencing. For each plant genotype, 20 independent top-strand clones were sequenced. For tested regions, bisulphite DNA sequences were analysed, and the levels of DNA methylation were calculated using the online Kismeth program^54^. For each genotype, the percentage of cytosine methylation in each context (CG, CHG or CHH) was calculated.

### Trypan blue staining

Leaves were immersed in 5 ml lactophenol trypan blue solution (250 μg ml^−1^ trypan blue, 25% (w/v) lactic acid, 25% water-saturated phenol, 25% glycerol, H_2_O), slow-release vacuum-infiltrated for 5 min, and then reinfiltrated for an additional 5 min. Samples were then heated over boiling water for 2 min and cooled before replacement of the lactophenol trypan blue solution with a chloral hydrate solution (25 g in 10 ml H_2_O) for destaining. After multiple exchanges of chloral hydrate solution, samples were equilibrated for several hours in 70% glycerol. For examination, leaves were mounted adaxial side up and examined using a microscope.

## Additional information

**Accession codes**: All original data sets have been deposited in the Gene Expression Omnibus database under accession number GSE62256.

**How to cite this article:** Yang, L. *et al*. Salicylic acid biosynthesis is enhanced and contributes to increased biotrophic pathogen resistance in *Arabidopsis* hybrids. *Nat. Commun.* 6:7309 doi: 10.1038/ncomms8309 (2015).

## Supplementary Material

Supplementary Figures and Tables Supplementary Figures 1-11, Supplementary Tables 1-5

Supplementary Dataset 1 Summary of the selected genes included in Figure 2

Supplementary Dataset 2 Summary of the original clones sequencing data of bisulfite sequencing
