# Supplementary material for: Salicylic acid biosynthesis is enhanced and contributes to increased biotrophic pathogen resistance in Arabidopsis hybrids
Source: Nat Commun. 2015 Jun 12;6:7309. doi: 10.1038/ncomms8309 (PMC4490401; doi:10.1038/ncomms8309)
Supplement: Supplementary Figures and Tables — Supplementary Figures 1-11, Supplementary Tables 1-5 [file ncomms8309-s1.pdf]

Supplementary Figures

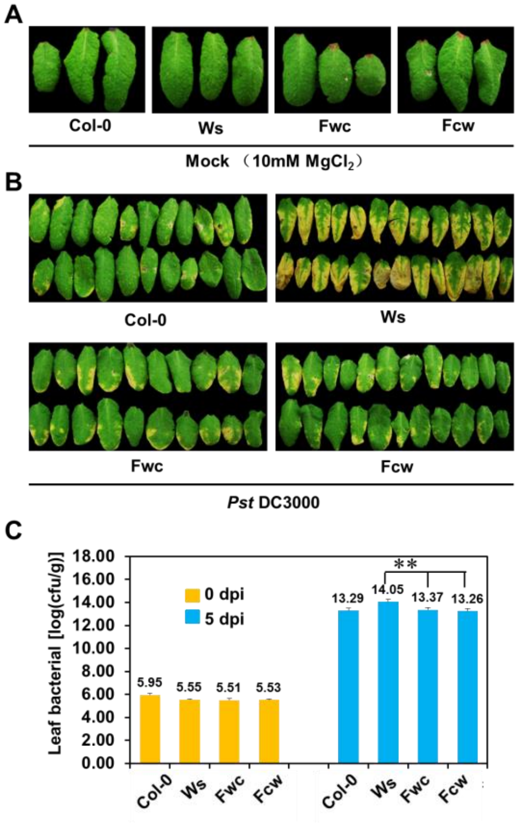

Supplementary Figure 1. Bacterial defence phenotypes of *Arabidopsis thaliana* F_1 hybrids and their parents.

(A) Phenotypes of F_1 hybrids and their parents 5 days post-infiltration (dpi) with $MgCl_2$ (10 mM). Fwc and Fcw, reciprocal F_1 hybrids, where maternal line is Ws and Col-0, respectively; (B) Phenotypes of F_1 hybrids and their parents 5 dpi with *Pseudomonas syringae* pv. *tomato* (*Pst*) DC3000 (1×10^5 cfu ml^{-1}); (C) Bacterial titre (log_{10}) of F_1 hybrids and their parents 5 dpi with *Pst* DC3000 (1×10^5 cfu ml^{-1}). $**p <$ 0.01 between hybrids and parents (Student's *t* test). Bacterial growth is expressed as mean values of viable bacteria per gram of leaf tissue ± SD. Error bars indicate SD. Data are shown as mean ± SD (n = 8, n means biological replication).

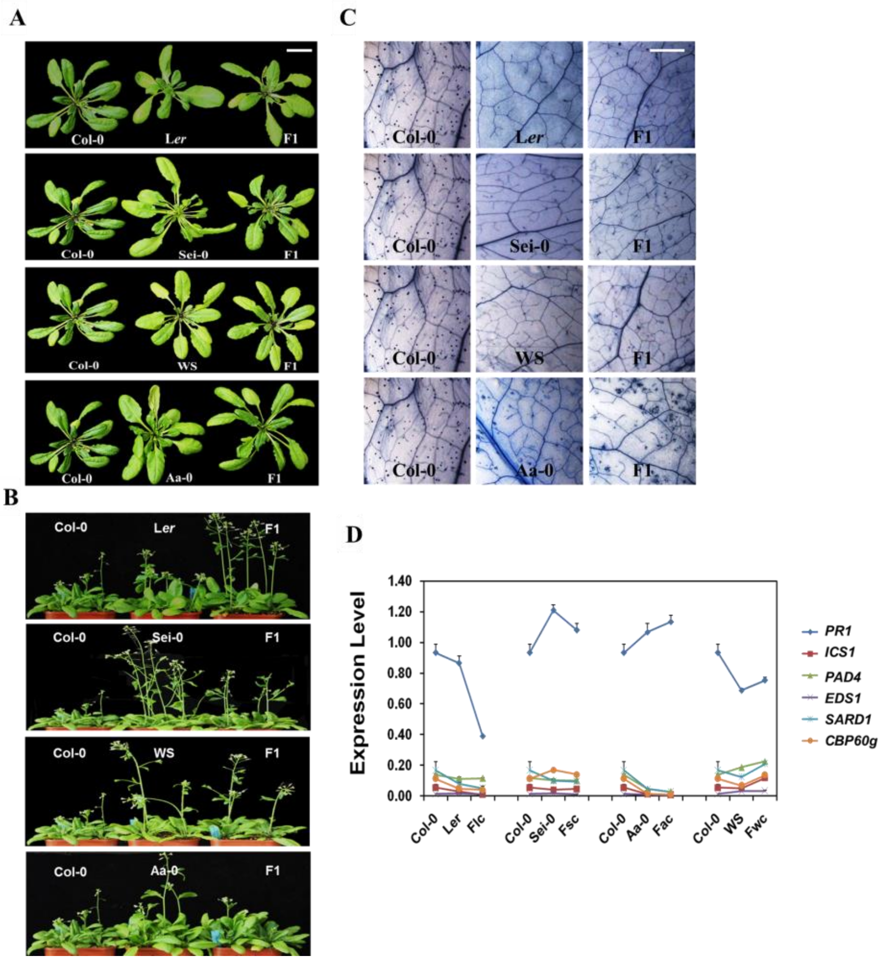

Supplementary Figure 2. The phenotype of hybrids from four combinations at 16 °C.

(A) Parents (left and middle) and F_1 hybrids (right) grown at 16 ºC. 6-weeks-old vegetative rosettes are shown. Scale bar (top) represents 3.5 cm. (B) Parents (left and middle) and F_1 hybrids (right) grown at 16 ºC. 6-weeks-old plants are shown. (C) Trypan Blue staining for dead cells in leaves of hybrids (right) and parents (left and middle). Scale bar (top right) represents 300 µm. (D) qPCR analyses of *PR1, ICS1, PAD4*, *EDS1*, *SARD1*, *SARD1*, *CBP60g* expression in F_1 hybrids and their parents at 16 ºC without pathogen inversion. Flc, F_1 where maternal line is L*er*. Fsc, F_1 where maternal line is Sei-0. Fac, F_1 where maternal line is Aa-0. Fwc, F_1 where maternal line is WS. Dates are standardized for abundance of the *Actin* transcript. The results are a representative of three biological repetitions. Error bars indicate SD. Data are shown as mean ± SD (n = 3, n means technical replication).

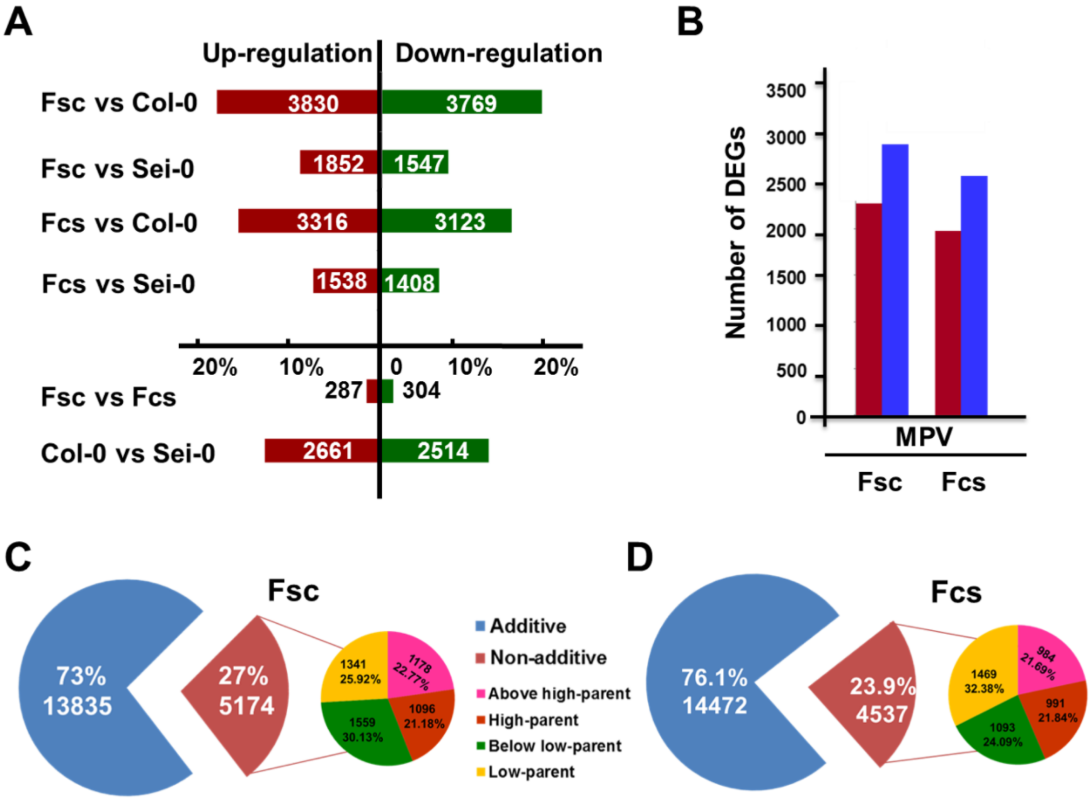

Supplementary Figure 3. Transcriptome analyses of hybrid and parental lines one day post infiltration with *Pst* DC3000.

(A) Statistical analyses of differentially expressed genes (DEGs) among inbred parents and their F_1 hybrids one day post infiltration with *Pst* DC3000. (B) Numbers of differentially expressed genes (DEGs) compared with mid-parent value (MPV) in each hybrid one day post infiltration with *Pst* DC3000. (C and D) Numbers of additively- and non-additively-expressed genes and the patterns of non-additively-expressed genes in each F_1 hybrid one day post infiltration with *Pst* DC3000.

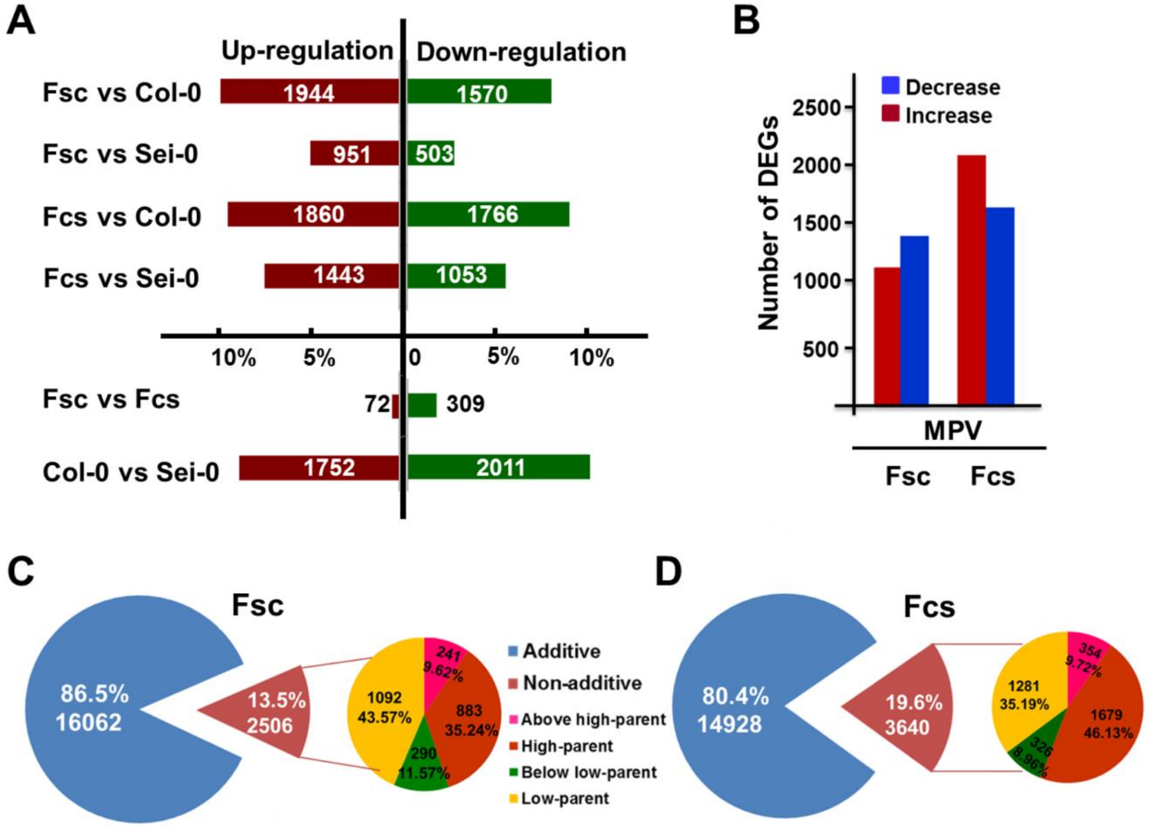

Supplementary Figure 4. Transcriptome analyses of hybrid and parental lines two days post infiltration with *Pst* DC3000.

(A) Statistical analyses of differentially expressed genes (DEGs) among inbred parents and their F_1 hybrids two days post infiltration with *Pst* DC3000. (B) Numbers of differentially expressed genes (DEGs) compared with MPV in each hybrid two days post infiltration with *Pst* DC3000. (C and D) Numbers of additively- and non-additively-expressed genes and the patterns of non-additively-expressed genes in each F_1 hybrid two days post infiltration with *Pst* DC3000.

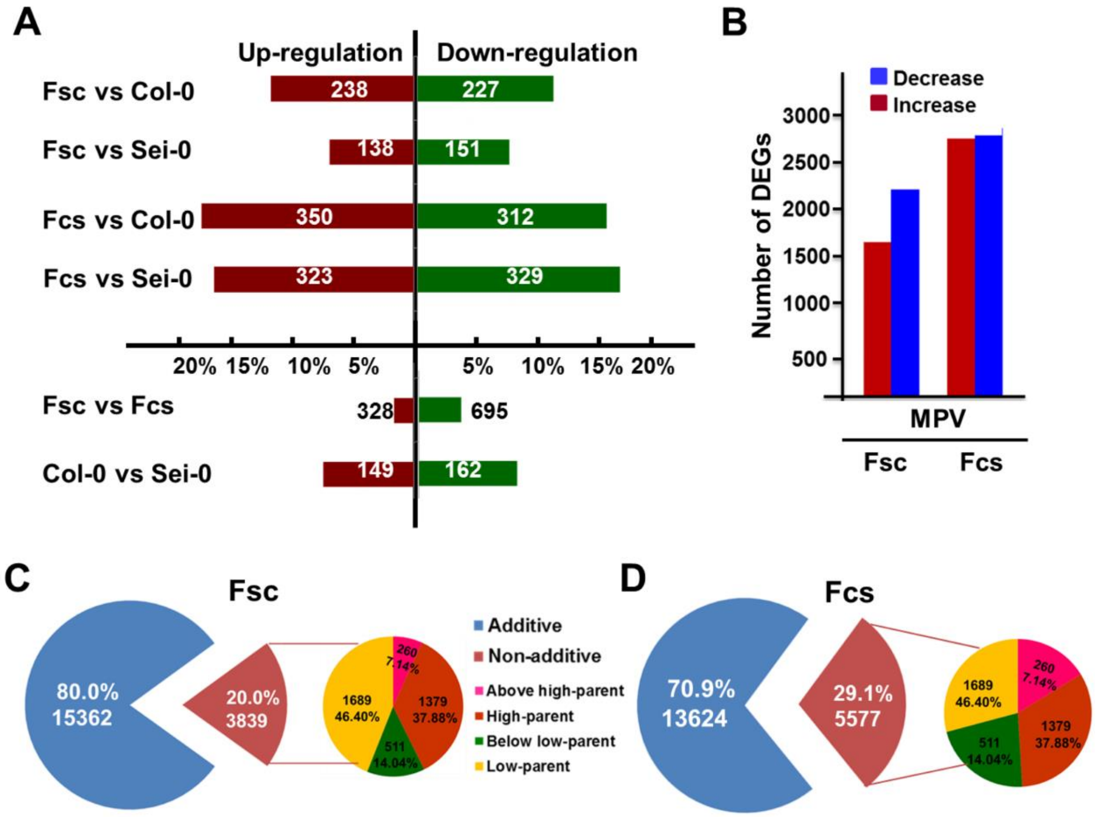

Supplementary Figure 5. Transcriptome analyses of hybrid and parental lines three days post infiltration with *Pst* DC3000.

(A) Statistical analyses of differentially expressed genes (DEGs) among inbred parents and their F_1 hybrids three days post infiltration with *Pst* DC3000. (B) Numbers of differentially expressed genes (DEGs) compared with MPV in each hybrid three days post infiltration with *Pst* DC3000. (C and D) Numbers of additively- and non-additively-expressed genes and the patterns of non-additively-expressed genes in each F_1 hybrid three days post infiltration with *Pst* DC3000.

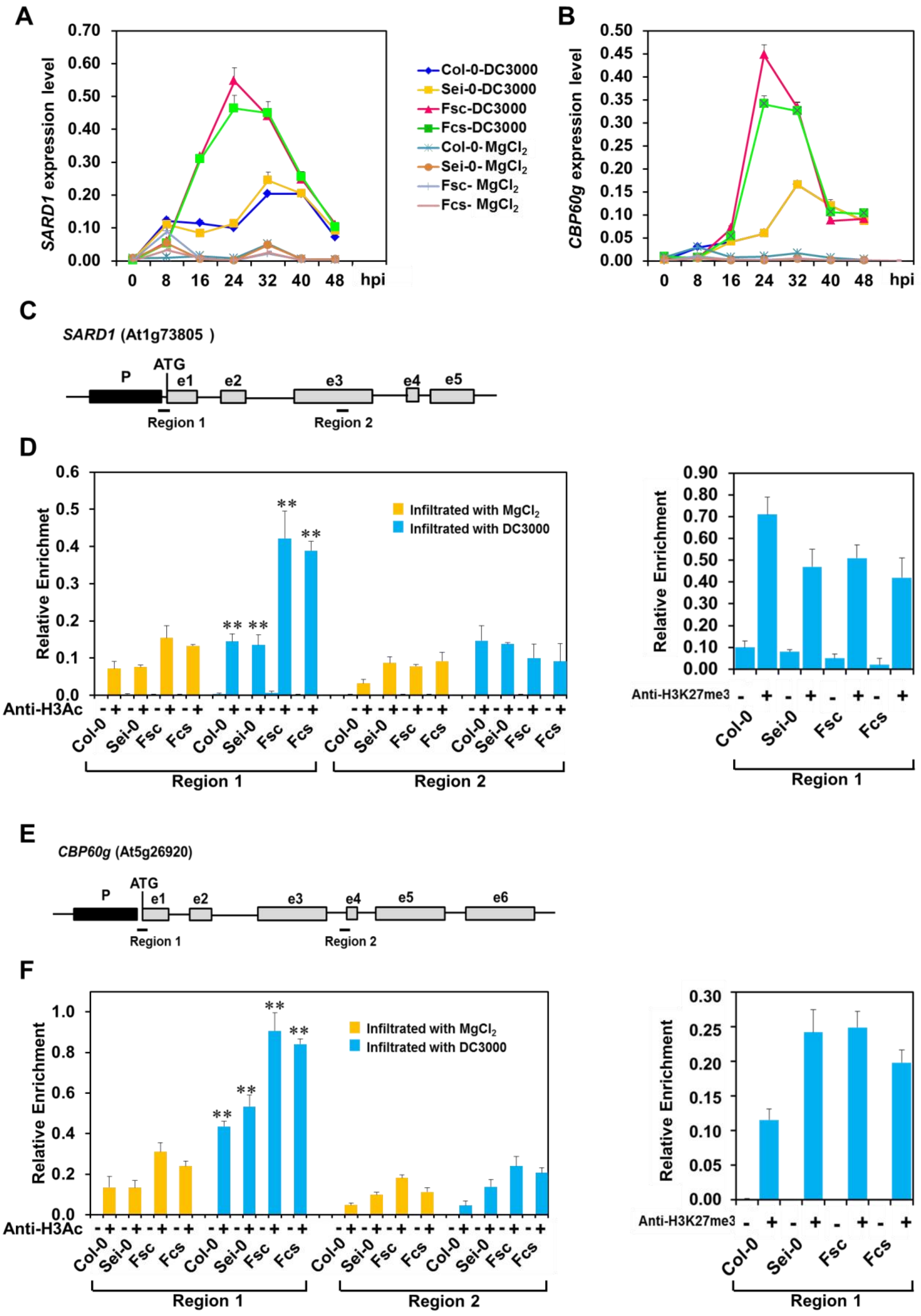
A
SARD1 expression level
hpi
Col-0-DC3000
Sei-0-DC3000
Fsc-DC3000
Fcs-DC3000
Col-0-MgCl2
Sei-0-MgCl2
Fsc- MgCl2
Fcs- MgCl2
B
CBP60g expression level
hpi
C
SARD1 (At1g73805)
P
ATG
e1
e2
e3
e4
e5
Region 1
Region 2
D
Relative Enrichment
Infiltrated with MgCl2
Infiltrated with DC3000
Anti-H3Ac
Col-0
Sei-0
Fsc
Fcs
Region 1
Region 2
Anti-H3K27me3
E
CBP60g (At5g26920)
P
ATG
e1
e2
e3
e4
e5
e6
Region 1
Region 2
F
Relative Enrichment
Infiltrated with MgCl2
Infiltrated with DC3000
Anti-H3Ac
Anti-H3K27me3
Region 1
Region 2

Supplementary Figure 6. Increased H3 acetylation correlated with altered expression of *CBP60g* and *SARD1* in *Arabidopsis thaliana* F_1 hybrids

(A and B) qPCR analyses of *SARD1* and *CBP60g* expression in F_1 hybrids and parents of Col-0 × Sei-0 every 8 h post-infiltration (hpi) up to 48 hpi with *Pseudomonas syringae* pv. *tomato* (*Pst*) DC3000 at 1×10^5 cfu ml^{-1} or $MgCl_2$. Fsc, F_1 where maternal line is Sei-0; Fcs, F_1 where maternal line is Col-0. Dates are standardized for abundance of the *Actin* transcript. (C and E) Regions of *SARD1* and *CBP60g* used for ChIP-qPCR assays. (D and F) ChIP-qPCR analyses of promoter fragments (region 1) and exon fragments (region 2) of *SARD1* and *CBP60g* in F_1 hybrids and their parents using anti-H3Ac antibody at 1 dpi with *Pst* DC3000 or $MgCl_2$, and ChIP-qPCR analyses of region 1 of *SARD1* and *CBP60g* in F_1 hybrids and their parents using anti-H3K27me3 antibody at 1 dpi with *Pst* DC3000. ChIP values were normalised to their respective DNA inputs. The results are a representative of three biological repetitions. Error bars indicate SD. Data are shown as mean ± SD (n = 3, n means technical replication). $^{**}p < 0.01$ between infiltrated with *Pst* DC3000 and $MgCl_2$ of respective samples (Student's *t* test).

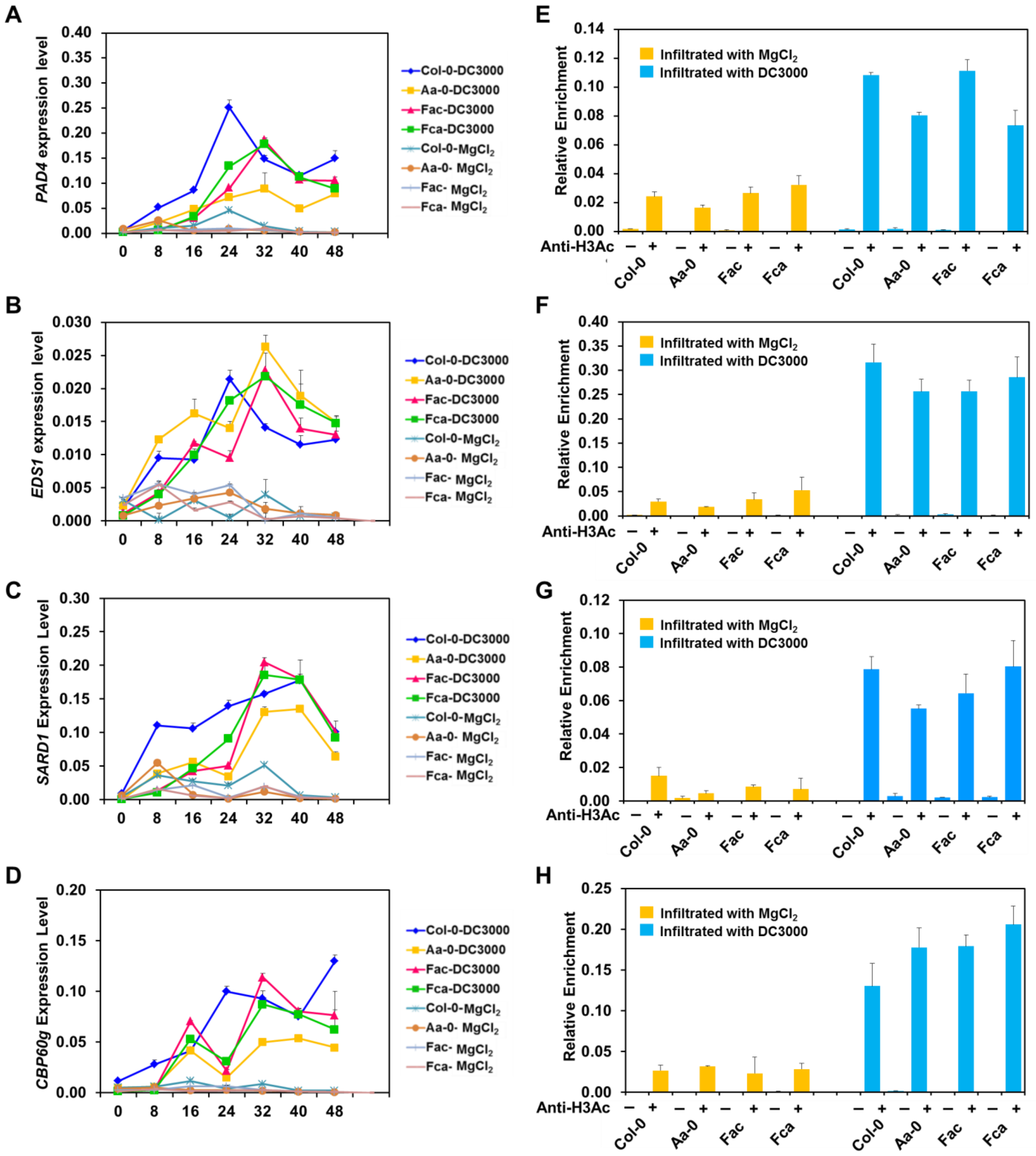

Supplementary Figure 7. Expression levels of the key genes in SA biosynthesis pathway are not up-regulated in hybrids of Col-0 × Aa-0.

(A to D) qPCR analyses of *PAD4, EDS1, SARD1* and *CBP60g* expression in F_1 hybrids and parents of Col-0 × Aa-0 every 8 h post-infiltration (hpi) up to 48 hpi with *Pseudomonas syringae* pv. *tomato* (*Pst*) DC3000 at 1×10^5 cfu ml^{-1} or $MgCl_2$. Fac, F_1 where maternal line is Aa-0; Fca, F_1 where maternal line is Col-0. Error bars indicate SD. The result is a representative of three biological repetitions. Dates are standardized for abundance of the *Actin* transcript. (E to H) ChIP-qPCR analyses of the promoter fragments (region 1) of *PAD4, EDS1, SARD1* and *CBP60g* in F_1 hybrids and parents of Col-0 × Aa-0 using anti-H3Ac antibody one day post infiltration with *Pst* DC3000 or $MgCl_2$. The ChIP values were normalized to their respective DNA inputs. The results are a representative of three biological repetitions. Error bars indicate SD. Data are shown as mean ± SD (n = 3, n means technical replication).

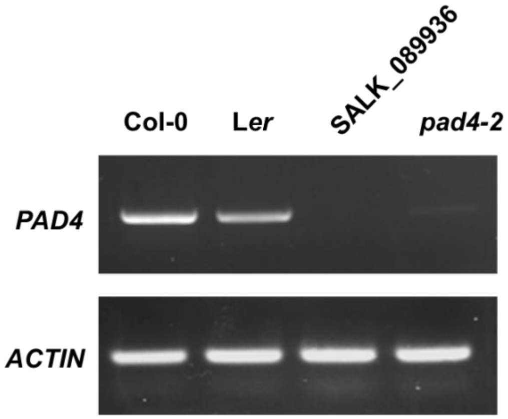

Supplementary Figure 8. ***PAD4*** **Expression in** ***pad4*** **mutants was abolished as revealed by RT-PCR assays.**

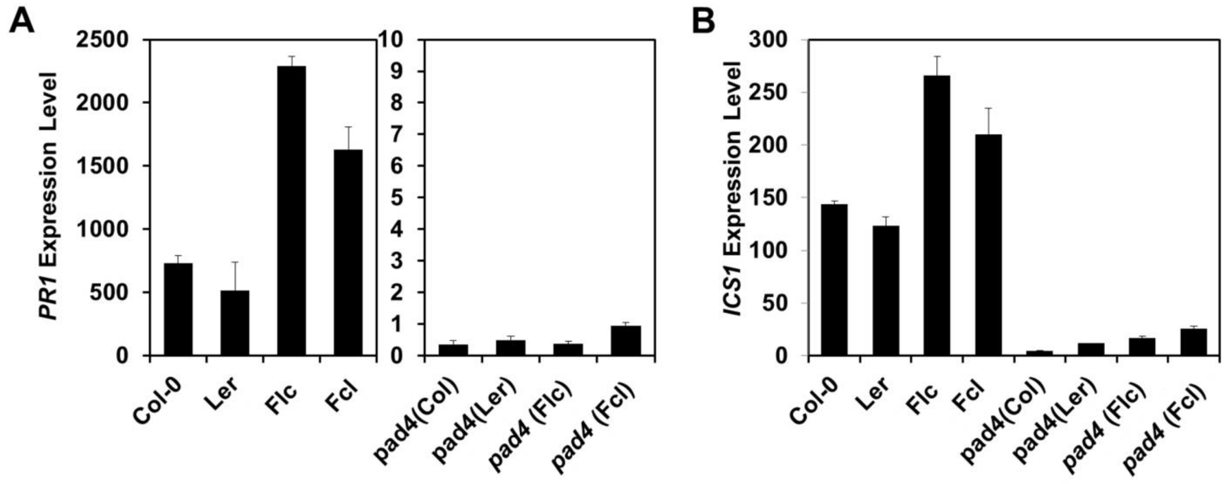

Supplementary Figure 9. Expression levels of *PR1* and *ICS1* in hybrids of Col-0 &L*er*, *pad4* (Col) & *pad4* (L*er*) combinations and their parents.

(A and B) qPCR analyses of *PR1* and *ICS1* expression in hybrids of Col-0 & L*er*, *pad4* (Col) & *pad4* (L*er*) combinations and their parents one day post infiltration with *Pst* DC3000 at 1×10^5 cfu ml^{-1}. Flc, F_1 where maternal line is L*er*; Fcl, F_1 where maternal line is Col-0. Data are standardized for abundance of *Actin* transcript. The results are a representative of three biological repetitions. Error bars indicate SD. Data are shown as mean ± SD (n = 3, n means technical replication).

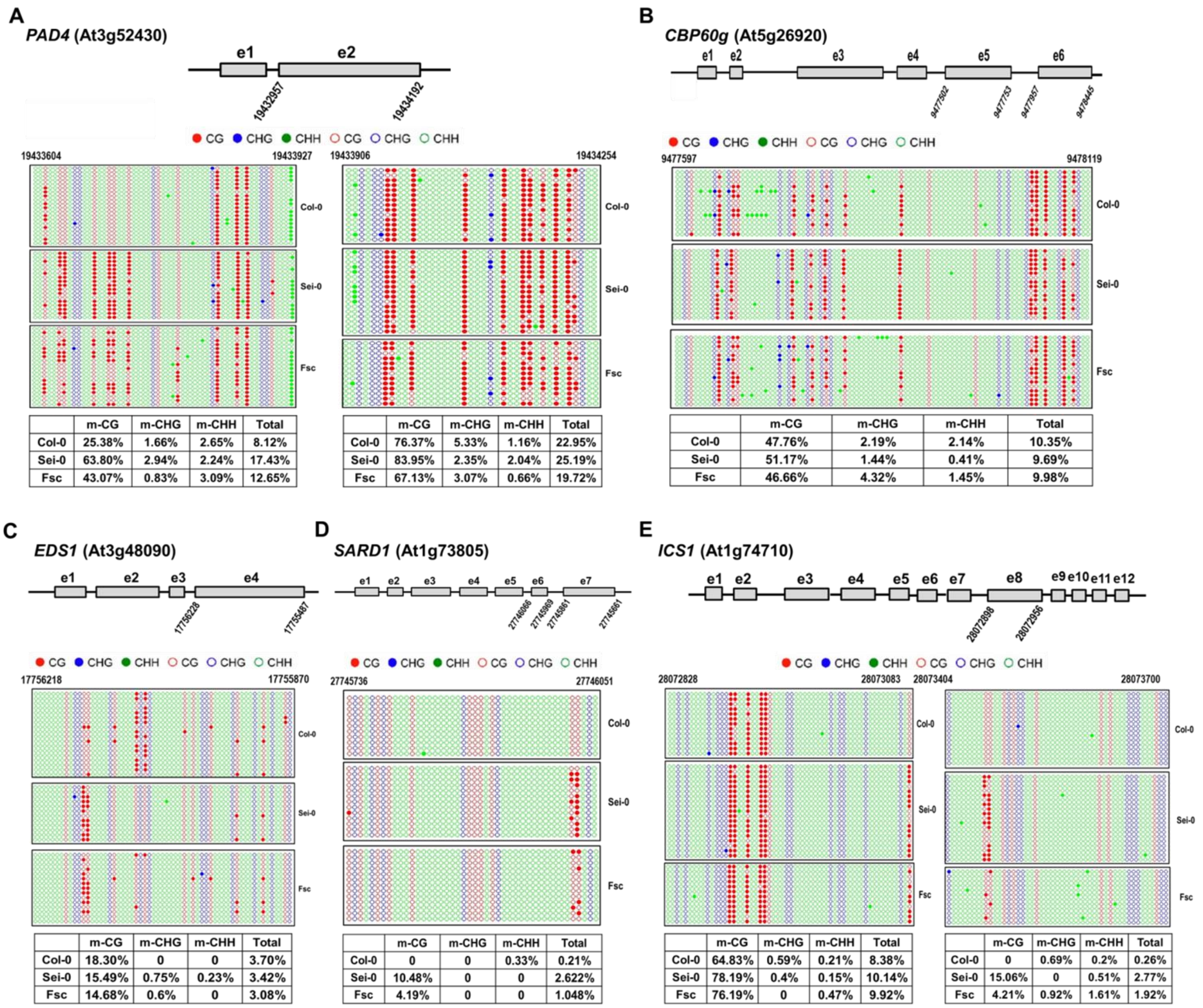

A, PAD4 (19433604–19433927)

	m-CG	m-CHG	m-CHH	Total
Col-0	25.38%	1.66%	2.65%	8.12%
Sei-0	63.80%	2.94%	2.24%	17.43%
Fsc	43.07%	0.83%	3.09%	12.65%

A, PAD4 (19433906–19434254)

	m-CG	m-CHG	m-CHH	Total
Col-0	76.37%	5.33%	1.16%	22.95%
Sei-0	83.95%	2.35%	2.04%	25.19%
Fsc	67.13%	3.07%	0.66%	19.72%

B, CBP60g (9477597–9478119)

	m-CG	m-CHG	m-CHH	Total
Col-0	47.76%	2.19%	2.14%	10.35%
Sei-0	51.17%	1.44%	0.41%	9.69%
Fsc	46.66%	4.32%	1.45%	9.98%

C, EDS1 (17756218–17755870)

	m-CG	m-CHG	m-CHH	Total
Col-0	18.30%	0	0	3.70%
Sei-0	15.49%	0.75%	0.23%	3.42%
Fsc	14.68%	0.6%	0	3.08%

D, SARD1 (27745736–27746051)

	m-CG	m-CHG	m-CHH	Total
Col-0	0	0	0.33%	0.21%
Sei-0	10.48%	0	0	2.622%
Fsc	4.19%	0	0	1.048%

E, ICS1 (28072828–28073083)

	m-CG	m-CHG	m-CHH	Total
Col-0	64.83%	0.59%	0.21%	8.38%
Sei-0	78.19%	0.4%	0.15%	10.14%
Fsc	76.19%	0	0.47%	9.92%

E, ICS1 (28073404–28073700)

	m-CG	m-CHG	m-CHH	Total
Col-0	0	0.69%	0.2%	0.26%
Sei-0	15.06%	0	0.51%	2.77%
Fsc	4.21%	0.92%	1.61%	1.92%

Supplementary Figure 10. DNA methylation levels of five genes in F_1 hybrids and their parents.

(A to E) Dot-plot analysis and percentage methylation levels of CG, CHG and CHH of *PAD4* (A), *CBP60g* (B), *EDS1* (C), *SARD1* (D) and *ICS1* (E) in Col-0 (top), Sei-0 (middle) and Fsc (bottom) in selected regions. Fsc, F_1 where maternal line is Sei-0. For each gene, a total of 13 to 20 individual gene body fragments were sequenced and analyzed. Red, blue and green circles indicate CG, CHG and CHH methylation (closed) or no methylation (open), respectively.

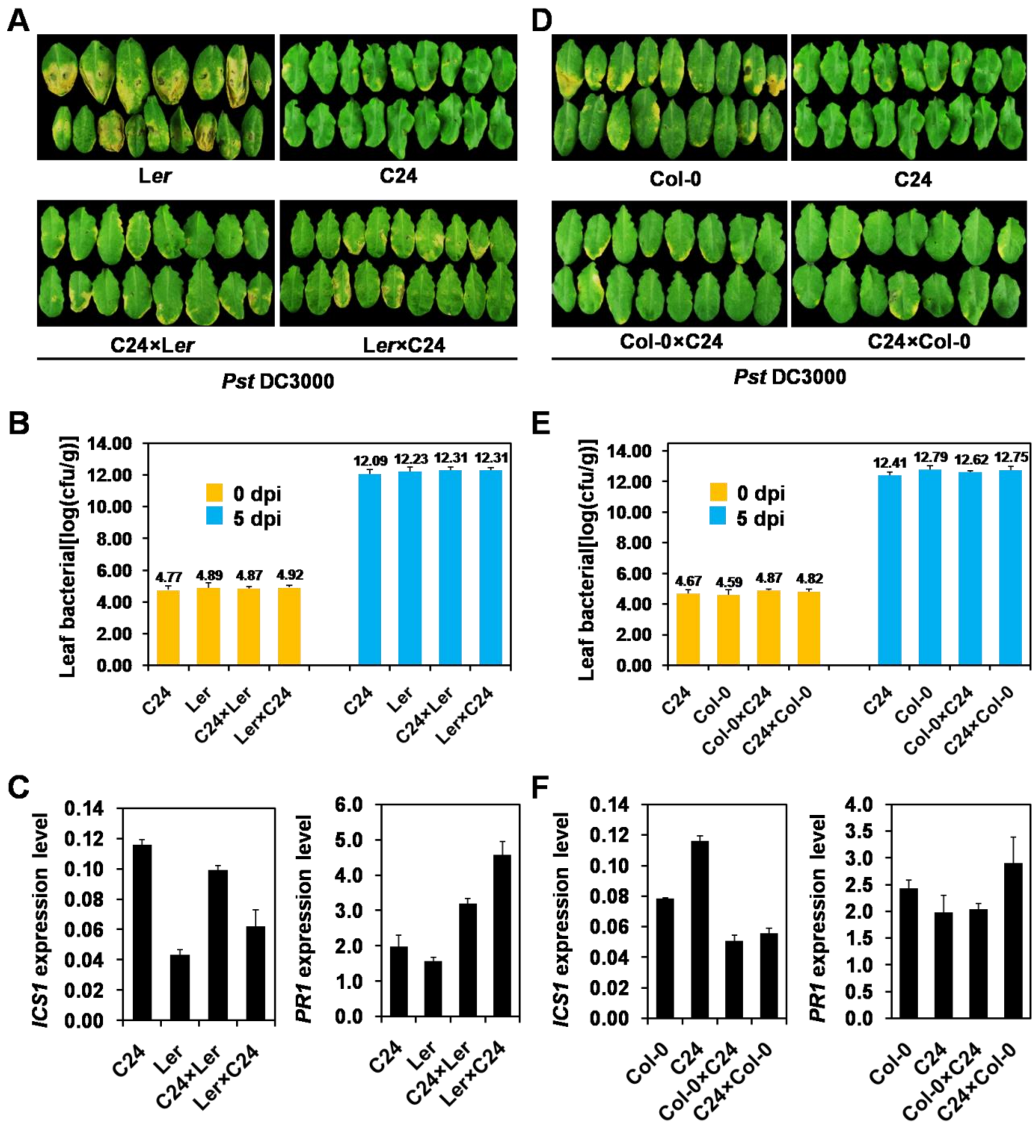

Supplementary Figure 11. No Direct Relationship between Heterosis for Biomass and for *Pst* DC3000 Defence in *Arabidopsis* Hybrids.

(A and D) Phenotypes of F_1 hybrids and their parents five days post infiltration with *Pst* DC3000 (1×10^5 cfu ml^{-1}). L*er* × C24, F_1 where maternal line is L*er*; C24 × L*er*, F_1 where maternal line is C24. (B and E) Bacterial titer (in $\log_{10}$) of hybrids and their parents five days post infiltration with *Pst* DC3000 (1×10^5 cfu ml^{-1}). Bacterial growth is expressed as mean values of viable bacteria per gram of leaf tissue ± SD. Error bars indicate SD. Data are shown as mean ± SD (n = 8, n means biological replication). (C and F)

qPCR analyses of *PR1* and *ICS1* expression in parental lines and their F_1 hybrids one day post infiltration (dpi) with *Pst* DC3000 (1×10^5 cfu ml^{-1}). Dates are standardized for abundance of the *Actin* transcript. The results are a representative of three biological repetitions. Error bars indicate SD. Data are shown as mean ± SD (n = 3, n means technical replication).

Supplementary Tables

Supplementary Table 1. Detection of heterosis for *Pst* DC3000 defence in *Arabidopsis* hybrid combinations.

Ecotype	Combination	Show heterosis for *Pst* DC3000 defence
Col-0		
**Sei-0**	**Col-0&Sei-0**	**YES**
**L*er***	**Col-0&L*er***	**YES**
WS	Col-0& WS	NO
Aa-0	Col-0& Aa-0	NO
No-0	Col-0& No-0	NO
Nd-0	Col-0& Nd-0	NO
Est-0	Col-0& Est-0	NO
C24	Col-0& Est-0	NO
Co-1	Col-0& Co-1	NO
Gr-1	Col-0& Gr-1	NO
Ha-0	Col-0& Ha-0	NO
Dr-0	Col-0& Dr-0	NO
Kil-0	Col-0& Kil-0	NO
Kro-0	Col-0& Kro-0	NO
Bch-1	Col-0& Bch-1	NO
Db-1	Col-0& Db-1	NO
Hs-0	Col-0& Hs-0	NO
An-1	Col-0& An-1	NO
La-0	Col-0& La-0	NO
Lip-0	Col-0& Lip-0	NO

Supplementary Table 2. Correlation analyses of different replicates in mRNA-seq.

			R2	R3				R2	R3
**Col-0**	**1dpi**	**R1**	0.946	0.983	**Sei-0**	**1dpi**	**R1**	0.983	0.940
		**R2**		0.973			**R2**		0.971
	**2dpi**	**R1**	0.980	0.910		**2dpi**	**R1**	0.910	0.883
		**R2**		0.929			**R2**		0.991
	**3dpi**	**R1**	0.948	0.960		**3dpi**	**R1**	0.721	0.858
		**R2**		0.838			**R2**		0.972
**Fsc**	**1dpi**	**R1**	0.919	0.953	**Fcs**	**1dpi**	**R1**	0.959	0.852
		**R2**		0.991			**R2**		0.895
	**2dpi**	**R1**	0.967	0.946		**2dpi**	**R1**	0.973	0.964
		**R2**		0.959			**R2**		0.954
	**3dpi**	**R1**	0.958	0.987		**3dpi**	**R1**	0.902	0.922
		**R2**		0.975			**R2**		0.957

R, replicate.

Supplementary Table 3. Analyses of mapped mRNA-seq reads in parents and hybrid lines.

		Aligned Bases (×10^6)			% of Gene Coverage		
		R1	R2	R3	R1	R2	R3
**Col-0**	**1dpi**	18.9	21.8	29.1	91.7	93.7	92.7
	**2dpi**	21.5	28.6	22.3	87.9	90.9	89.9
	**3dpi**	19.2	31.1	51.3	90.8	91.9	93.1
**Sei-0**	**1dpi**	17.8	27.8	45.6	85.8	87.6	87.8
	**2dpi**	24.4	30.9	21.7	86.6	84.2	87.1
	**3dpi**	25.7	18.1	15.1	86.6	87.7	88.1
**Fsc**	**1dpi**	32.9	11.5	38.2	89.4	90.3	88.3
	**2dpi**	26.4	28.1	35.9	87.5	88.7	90.7
	**3dpi**	19.2	18.2	26.4	87.8	89.5	90.8
**Fcs**	**1dpi**	8.2	17.2	21.5	90.8	88.9	91.2
	**2dpi**	31.6	17.1	20.6	88.7	86.2	90.5
	**3dpi**	44.0	12.4	20.8	90.8	89.5	91.6

Supplementary Table 4. Biomass Phenotypes Measured for Parental Lines and their Reciprocal Hybrids.

No. of rosette leaves											
	**MPH**	**BPH**		**MPH**	**BPH**		**MPH**	**BPH**		**MPH**	**BPH**
**Fsc**	-4.7%	-7.8%	**Flc**	17.3%	1.9%	**Fec**	-14.1%	-16.1%	**Fac**	5.5%	4.8%
**Fcs**	0.6%	-2.7%	**Fcl**	16.8%	1.5%	**Fce**	-9.6%	-11.7%	**Fca**	6.7%	6.0%
**Diameter of rosette leaves**											
**Fsc**	7.1%	-1.4%	**Flc**	22.7%	6.1%	**Fec**	-11.4%	-13.1%	**Fac**	1.9%	-3.0%
**Fcs**	2.1%	-6.1%	**Fcl**	20.9%	4.6%	**Fce**	-1.7%	-3.6%	**Fca**	9.0%	3.8%
**Plant fresh weight**											
**Fsc**	-9.1%	-19.4%	**Flc**	22.0%	2.9%	**Fec**	-41.7%	-41.7%	**Fac**	12.5%	12.5%
**Fcs**	1.8%	-19.7%	**Fcl**	23.6%	-2.9%	**Fce**	-12.5%	-12.5%	**Fca**	25.0%	25.0%

Fsc, F_1 where maternal line is Sei-0; Flc, F_1 where maternal line is L*er*; Fec, F_1 where maternal line is Est-0; Fac, F_1 where maternal line is Aa-0; Fcs, Fcl, Fce and Fca, F_1 where maternal lines are all Col-0. MPH = 1-(F_1/MPV). MPV, mid-parent value. BPH = 1-(F_1/BPV). BPV, best-parent value. Plants grown under short-day conditions (8 h light/16 h dark) for 30 days were selected for measurement (n = 40).

Supplementary Table 5. Summary of primers.

**Primers for ChIP (5'-3')**	
PAD4-CF-p	GATCACATGCTTTGATTCGC
PAD4-CR-p	GTCGTCTTCTTCAAAGTCTC
PAD4-CF-c	GGCTAAGCTTGAGCAAGCAA
PAD4-CR-c	CGAGTTCTTCGCTTTAACATCC
EDS1-CF-p	GTCCACTAAAGAAAAGAGAA
EDS1-CR-p	AAGATTACGACTGCTCCTGC
EDS1-CF-c	CTTAGGGTTACAGCTAGAAG
EDS1-CR-c	CAAGCATCCCTTCTAATGTC
CBP60g-CF-p	TCACTGCTGCTTCGTCAATAC
CBP60g-CR-p	CTAGGGCTGTTCCGAATCTTCATTG
CBP60g-CF-c	AGTGATGAGGTTTGGAGACTAGA
CBP60g-CR-c	ACGTTGTGTAACTCATTTCG
SARD1-CF-p	GATTATTCGCGTGGATCAGACTTCG
SARD1-CR-p	CTGCCATGGAATTGTTCTGGTGA
SARD1-CF-c	TAGACATCGTTGCTCTTCAC
SARD1-CR-c	CTCGAATTGTCTGTGAACAC
**Primers for Real-time PCR (5'-3')**	
PAD4-QF	ACCGAGGAACATCAGAGGTAC
PAD4-QR	AAATTCGCAATGTCGAGTGGC
EDS1-QF	CAAGAATCTTGAAGCTGTCATTGATC
EDS1-QR	TGTCCTGTGAACACTATCTGTTTTCTACT
ICS1-QF	GAACTCAAATCTCAACCTCC
ICS1-QR	ACTGCGACGAGAGAAGAAAC
SARD1-QF	CCTCAACCAGCCCTACGTTA
SARD1-QR	TAGTGGCTCGCAGCATATTG
CBP60g-QF	AATAACGAGGAGGATGAGAACG
CBP60g-QR	TCAGACACGGTAAGAAACATCG
Actin-QF	GGTAACATTGTGCTCAGTGGTGG
Actin-QR	GGTGCAACGACCTTAATCTTCAT
PR1-QF	CTCATACACTCTGGTGGG
PR1-QR	TTGGCACATCCGAGTC
**Primers for Promoter Sequencing (5'-3')**	
PR1-Pmt-F	GGTTTATGATCGGAACTTAGAG
PR1-Pmt-R	AGAGCACCTACAAGAGCTACA
ICS1-pmt-F	GACCAGGTTGAGTAAATCAGACA
ICS1-pmt-R	GTGAAGCCATTGCAGAAATTCGT
PAD4-pmt-F	CATGTAACTGATGAATCCATGTG
PAD4-pmt-R	TCGAATCGACAATCGTCCATGA
EDS1-pmt-F	ATGAAAGCTGAGGAAAGAGCT
EDS1-pmt-R	AGAGCTTCAAACGCCATTGAT
CBP60g-pmt-F	ATCGACGTGTACACTTCTCCCACAT
CBP60g-pmt-R	CTAGGGCTGTTCCGAATCTTCATTG

SARD1-pmt-F	AGTGGATGGCAATTCAGAAATGG
**Primers for F_1 Identification (5'-3')**	
NAG139-F	GGTTTCGTTTCACTATCCAGG
NAG139-R	AGAGCTACCAGATCCGATGG
CIW11-F	CCCCGAGTTGAGGTATT
CIW11-R	GAAGAAATTCCTAAAGCATTC
NGA6-F	ATGGAGAAGCTTACACTGATC
NGA6-R	TGGATTTCTTCCTCTCTTCAC
**Primers for Bisulfite Sequencing (5'-3')**	
PAD4M-F1	TAAGATTYGAGATTTAATGGTA
PAD4M-R1	ACCTTTTARATCTATTCCCCT
PAD4M-F2	AGGGGAATAGATYTAAAAGGT
PAD4M-R2	CAATTTCATTTTCATAACCCATT
EDS1M-F	AAAAATGGGTATTATGATTTTTTT
EDS1M-R	CCAAAATACATCTTCTACAATC
ICS1M-F1	AAGTGTTTTGAATAATTATAGAAT
ICS1M-R1	TCCAATACTATTATCTCACCTC
ICS1M-F2	AGATGTAGAGATTAAGAATTATG
ICS1M-R2	CCAACTACTATCCCTATCCC
CBP60gM-F	TAGATTAATTAAAGGTYGAAG
CBP60gM-R	CACTTACTTTAAAACTATTTC
SARD1M-F	GAAAGGGTTTATATGATTTTGA
SARD1M-R	TATCAACATAAACACCATTTCA
